# Supplementary material for: Systematic variation of the acceptor electrophilicity in donor-acceptor-donor emitters exhibiting efficient room temperature phosphorescence suited for digital luminescence
Source: Commun Chem. 2025 Sep 10;8:274. doi: 10.1038/s42004-025-01620-0 (PMC12423317; doi:10.1038/s42004-025-01620-0)
Supplement: Supplementary file 2 — Supplementary Information [file 42004_2025_1620_MOESM2_ESM.pdf]

Supplementary Information

Systematic variation of the acceptor electrophilicity in donor-acceptor-donor emitters exhibiting efficient room temperature phosphorescence suited for digital luminescence

Uliana Tsiko[1], Jannis Fidelius[2], Sebastian Kaiser[1], Heidi Thomas[1], Yana Bui Thi[1], Jan J. Weigand[2], Juozas V. Grazulevicius[3], Karl Sebastian Schellhammer[1], and Sebastian Reineke[1]*

[1]Dresden Integrated Center for Applied Physics and Photonic Materials (IAPP), Technische Universität Dresden, Hermann-Krone-Bau, Nöthnitzer Str. 61, 01187 Dresden, Germany

[2]Chair of Inorganic Molecular Chemistry, Faculty of Chemistry and Food Chemistry, Technische Universität Dresden, 01069 Dresden, Germany

[3]Department of Polymer Chemistry and Technology, Kaunas University of Technology, Radvilenu pl. 19, LT-50254, Kaunas, Lithuania

Table of Contents

S1 Synthesis of materials 2

S1.1 General procedure of Suzuki-Miyaura cross-coupling reactions 2

S1.2 Synthesis and verification of Py-2TA 3

S1.3 Synthesis and verification of PyCN-2TA 6

S1.4 Synthesis and verification of Pm-2TA 9

S1.5 Synthesis and verification of PmCN-2TA 13

S2 Crystallographic details 17

S3 Thermal properties

S4 Further results from materials simulations 18

S5 Molecular geometries 19

S5.1 BP-2TA 19

S5.2 Py-2TA 20

S5.3 PyCN-2TA 22

S5.4 Pm-2TA 23

S5.5 PmCN-2TA 24

S6 Further results from experimental photophysical characterization 43

S7 Characterization of programmable luminescent tags 49

S1 Synthesis of materials

S1.1 General procedure of Suzuki-Miyaura cross-coupling reactions

The target compounds were synthesized *via* Suzuki-Miyaura cross-coupling reactions by using bromide precursor (1 eq.) thianthrene-1-boronic acid (2.3 eq.), aqueous potassium carbonate (6.0 eq.) as a base and tetrakis(triphenylphosphine)palladium(0) (0.05 eq.) as catalyst. All dry reagents were added to a Schlenk flask and the atmosphere was purged with nitrogen for 30 min. After that dry tetrahydrofuran (20 ml) was added and, additionally, a solution of potassium carbonate (6 eq.) dissolved in 5 ml of water was added dropwise. The reaction mixture was heated up to 75 °C and stirred for 24 hours under nitrogen atmosphere. After cooling down, the mixture was poured into water. Dichloromethane was used for extraction. The organic phase was washed with brine and afterwards dried with sodium sulfate (Na_2SO_4). The solvent was evaporated. The crude product was purified by column chromatography (hexane/ethyl acetate (7/1) as eluent) and recrystalized from the eluent.

S1.2 Synthesis and verification of Py-2TA

3,5-di(thianthren-1-yl)pyridine (**Py-2TA**) was synthesized in accordance with the general procedure from 3,5-dibromopyridine (0.50 g, 2.11 mmol), thianthrene-1-boronic acid (1.26 g, 4.85 mmol), tetrakis(triphenylphosphine)palladium(0) (0.12 g, 0.10 mmol) and potassium carbonate (1.74 g, 12.6 mmol). The pure product was isolated as colorless crystals with 60 % yield (0.65 g, 1.28 mmol).

^{1}H NMR (500 MHz, CD_2Cl_2) δ 8.72 (d, *J* = 2.1 Hz, 2H, Py), 7.85 (t, *J* = 2.1 Hz, 1H, Py), 7.61 (dd, *J* = 5.9, 3.2 Hz, 2H, Ar), 7.53 (dd, *J* = 7.6, 1.1 Hz, 2H, Ar), 7.46 (dd, *J* = 7.7, 1.1 Hz, 2H, Ar), 7.40 – 7.37 (m, 4H, Ar), 7.28 (td, *J* = 7.5, 1.3 Hz, 2H, Ar), 7.23 (td, *J* = 7.5, 1.3 Hz, 2H, Ar).
^{13}C NMR ^{13}C NMR (126 MHz, CD_2Cl_2) δ 149.09, 138.55, 137.55, 136.38, 136.18, 135.34, 135.27, 135.15, 129.50, 128.99, 128.95, 128.57, 128.06, 127.80, 127.56.
HR-MS (m/z): calculated for $C_{29}H_{17}NS_4$ [M] = 507.70, found $([M]+H)^+$ = 508.03

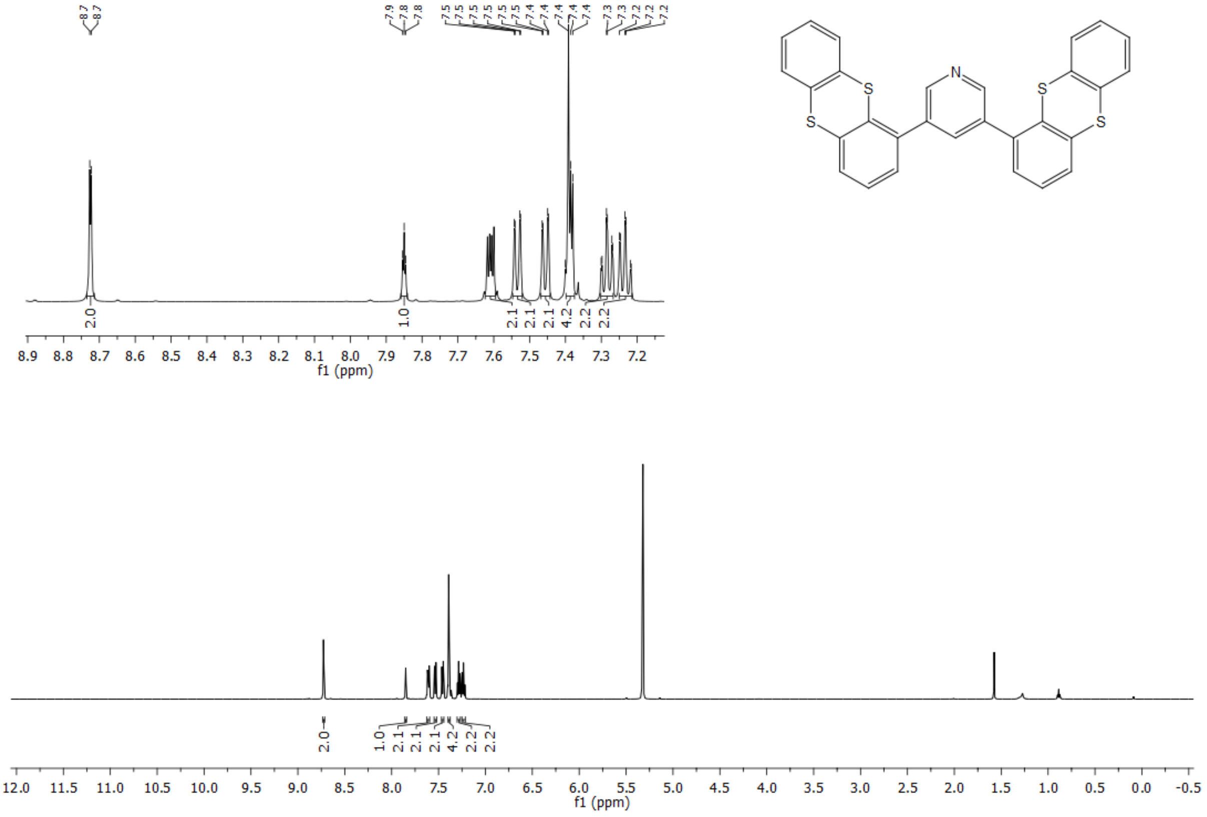

Figure S1. ^{1}H NMR spectrum of **Py-2TA** in CD_2Cl_2.

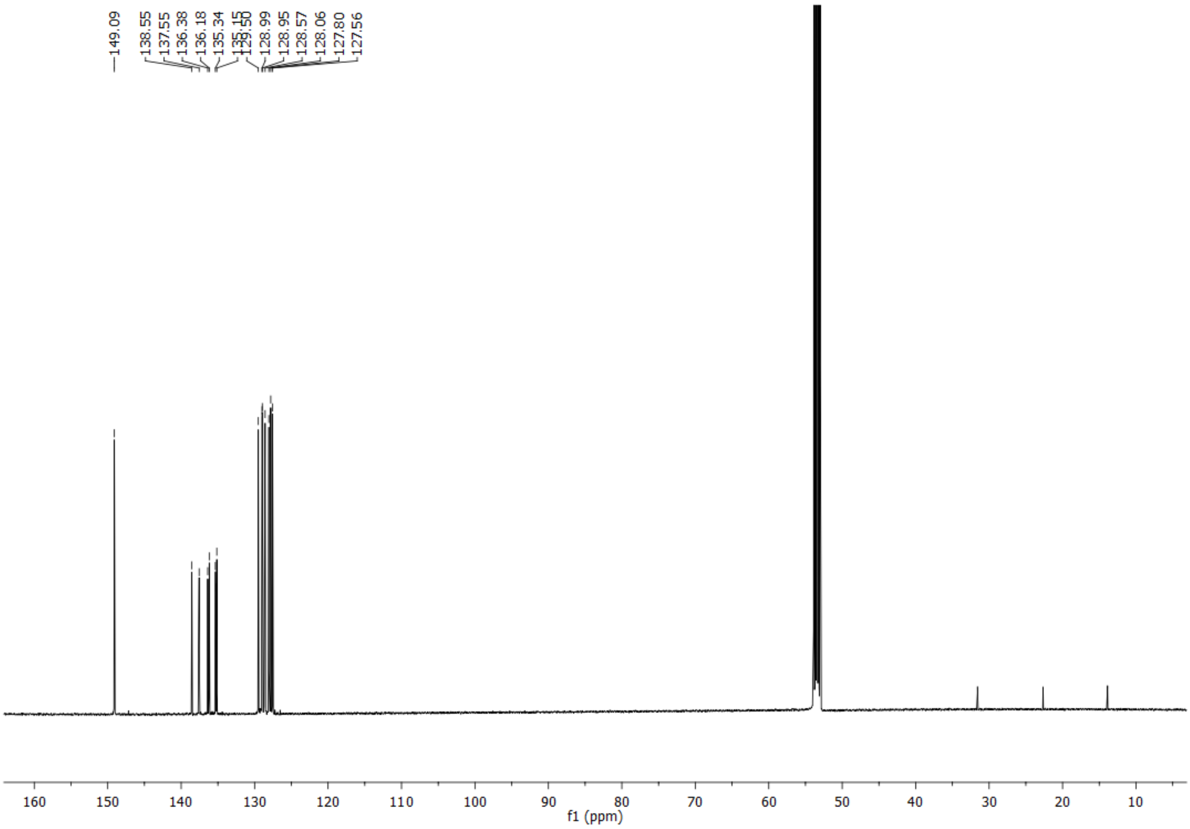

Figure S2. ^{13}C NMR spectrum of **Py-2TA** in CD_2Cl_2.

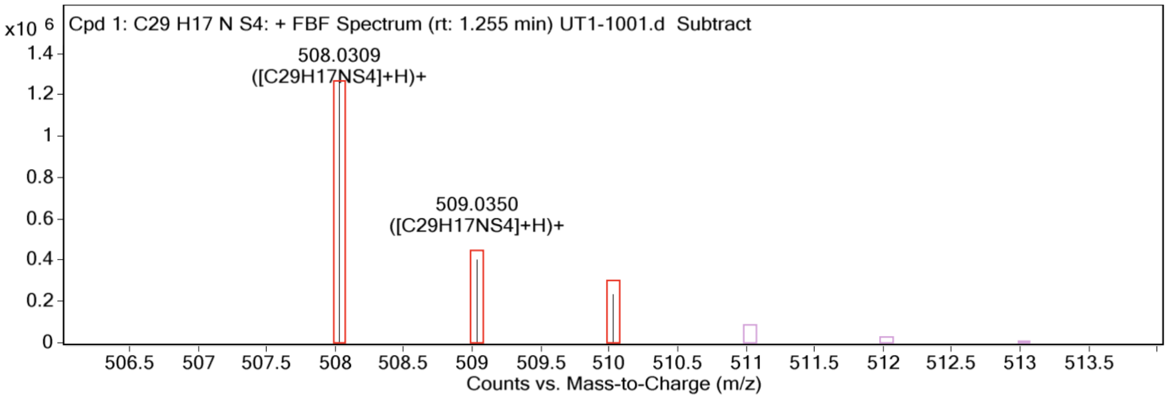

Figure S3. HRMS spectrum of **Py-2TA**.

Chromatogram

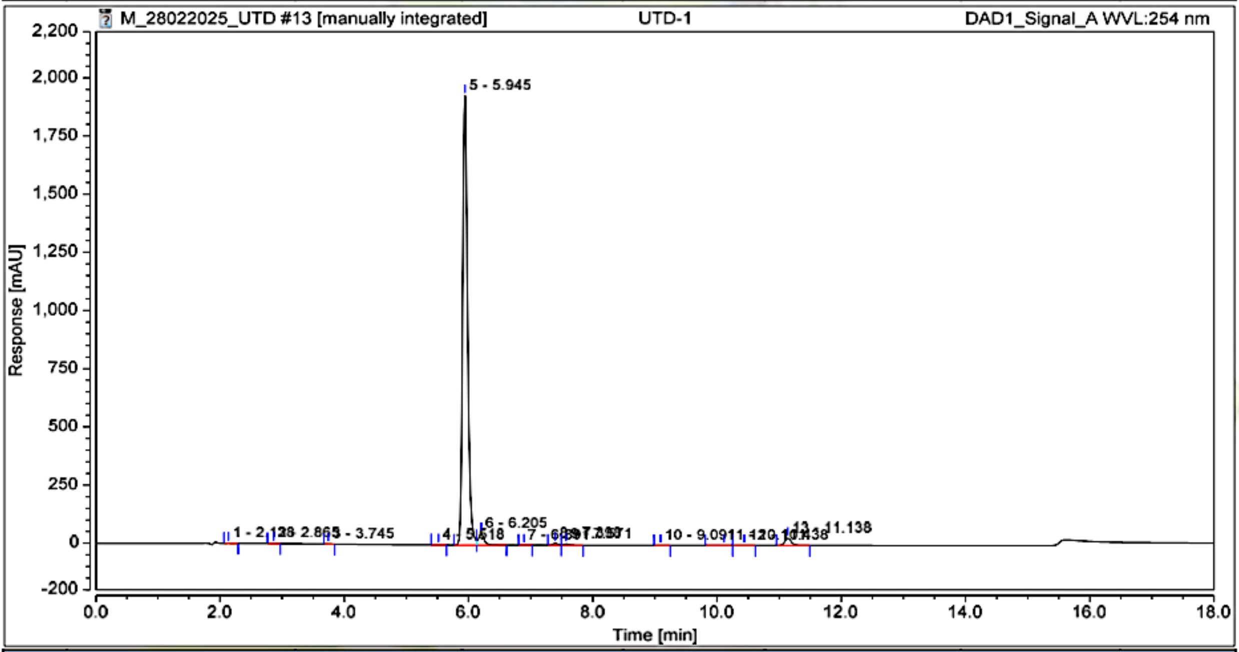

Integration Results

No.	Peak Name	Retention Time min	Area mAU*min	Height mAU	Relative Area %	Relative Height %	Amount n.a.
1		2.138	0.031	0.453	0.02	0.02	n.a.
2		2.865	0.089	1.081	0.05	0.05	n.a.
3		3.745	0.051	0.645	0.03	0.03	n.a.
4		5.518	0.054	0.642	0.03	0.03	n.a.
5		5.945	177.809	1930.152	94.35	95.12	n.a.
6		6.205	5.202	50.038	2.76	2.47	n.a.
7		6.891	0.046	0.502	0.02	0.02	n.a.
8		7.398	0.705	8.049	0.37	0.40	n.a.
9		7.571	0.533	5.510	0.28	0.27	n.a.
10		9.091	0.032	0.295	0.02	0.01	n.a.
11		10.111	0.134	0.551	0.07	0.03	n.a.
12		10.438	0.019	0.161	0.01	0.01	n.a.
13		11.138	3.750	31.056	1.99	1.53	n.a.
**Total:**			**188.455**	**2029.137**	**100.00**	**100.00**	

Figure S4. HPLC chromatogram and peak description of **Py-2TA**.

S1.3 Synthesis and verification of PyCN-2TA

3,5-di(thianthren-1-yl)-4-cyanopyridine (**PyCN-2TA**) was obtained according to the general procedure from 3,5-dibromo-4-pyridinecarbonitrile (0.20 g, 0.76 mmol), thianthrene-1-boronic acid (0.46 g, 1.77 mmol), tetrakis(triphenylphosphine)palladium(0) (0.04 g, 0.03 mmol) and potassium carbonate (0.63 g, 4.45 mmol). The pure product was isolated as light-yellow powder with 66 % yield (0.27 g, 0.51 mmol).

^{1}<u>H NMR</u> (500 MHz, CD_2Cl_2) δ 8.81 (d, *J* = 17.3 Hz, 2H, Py), 7.72 – 7.69 (m, 2H, Ar), 7.56 (t, *J* = 6.6 Hz, 2H, Ar), 7.50 – 7.47 (m, 2H, Ar), 7.44 (t, *J* = 7.4 Hz, 2H, Ar), 7.39 – 7.28 (m, 4H, Ar), 7.25 (dt, *J* = 15.5, 7.8 Hz, 2H, Ar).

^{13}<u>C NMR</u> (126 MHz, CD_2Cl_2) δ 150.48, 150.30, 137.58, 137.20, 135.89, 134.74,130.02, 129.18, 128.63, 128.25, 127.96, 127.76, 121.50, 121.00, 114.64, 114.52.

<u>HR-MS (m/z):</u> calculated for $C_{30}H_{16}N_2S_4$ [M] = 532.71, found $([M]+H)^{+}$ = 533.03

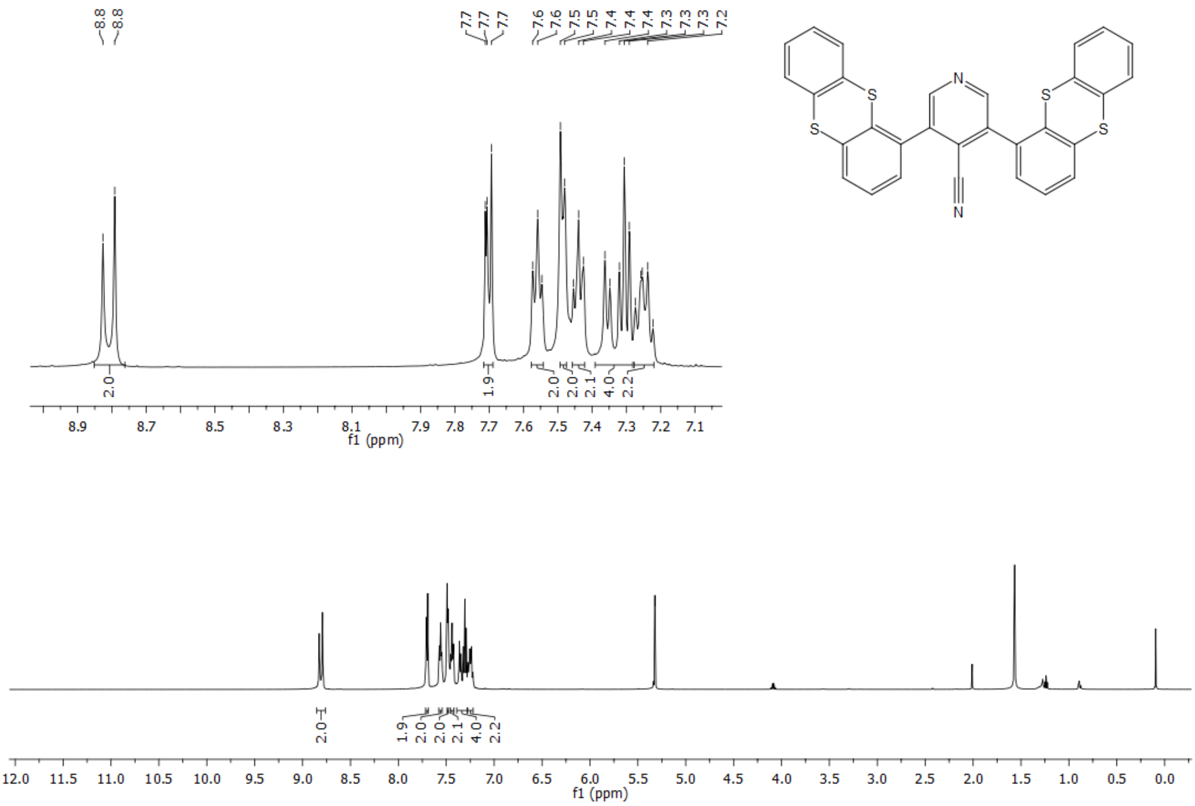

Figure S5. ^{1}H NMR spectrum of **PyCN-2TA** in CD_2Cl_2.

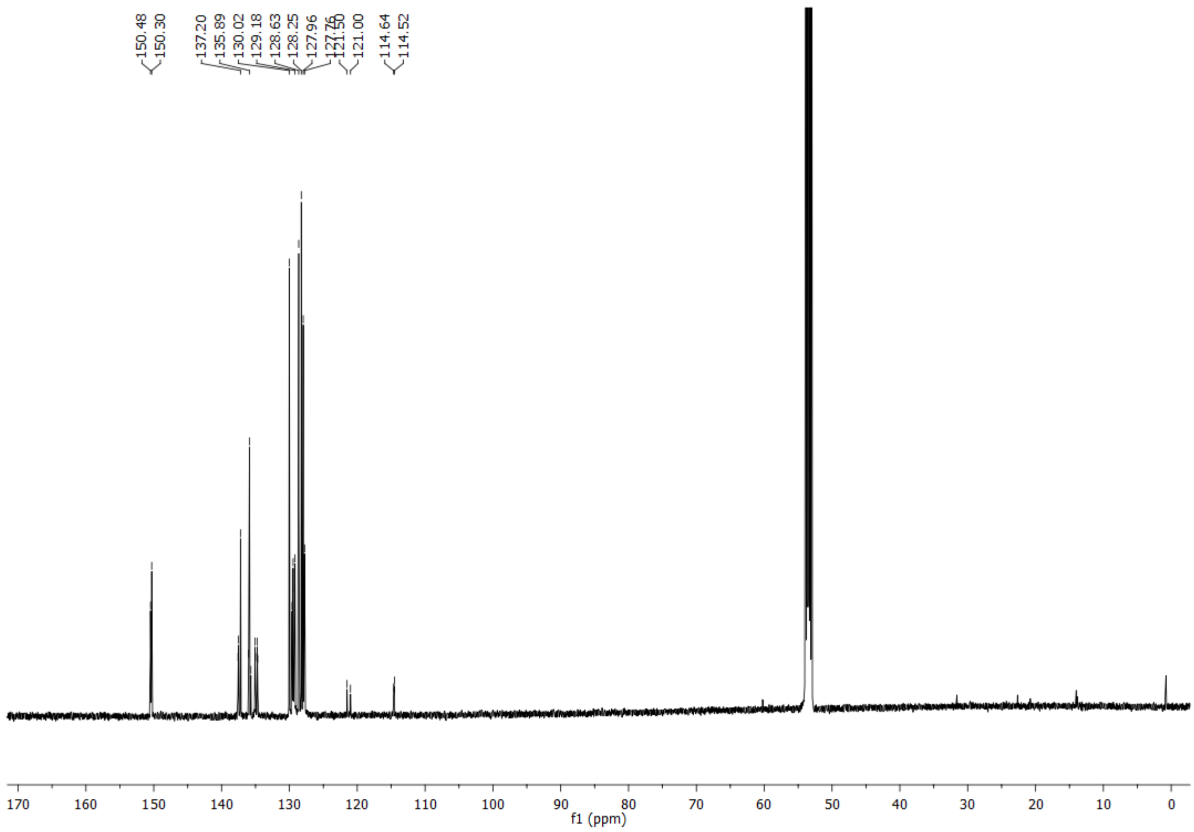

Figure S6. ^{13}C NMR spectrum of **PyCN-2TA** in CD_2Cl_2.

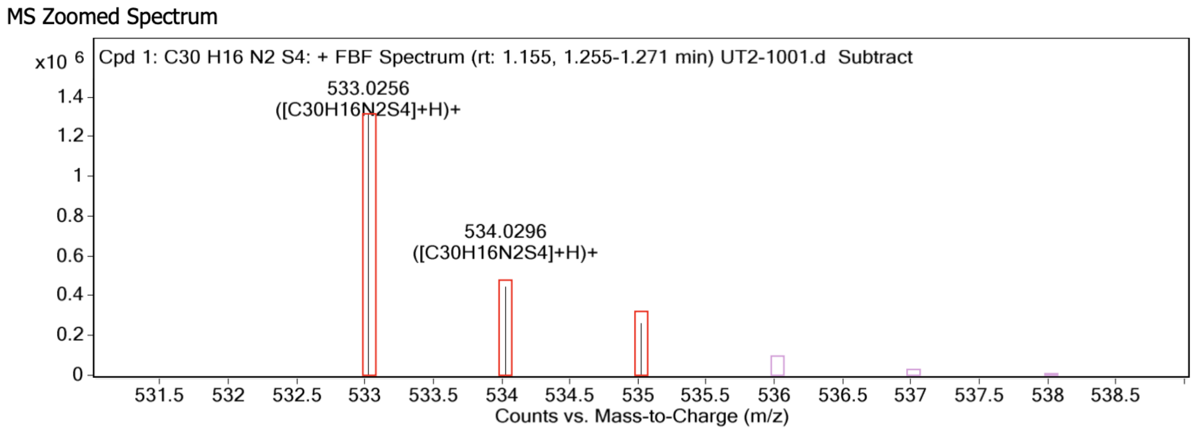

Figure S7. HRMS spectrum of **PyCN-2TA**.

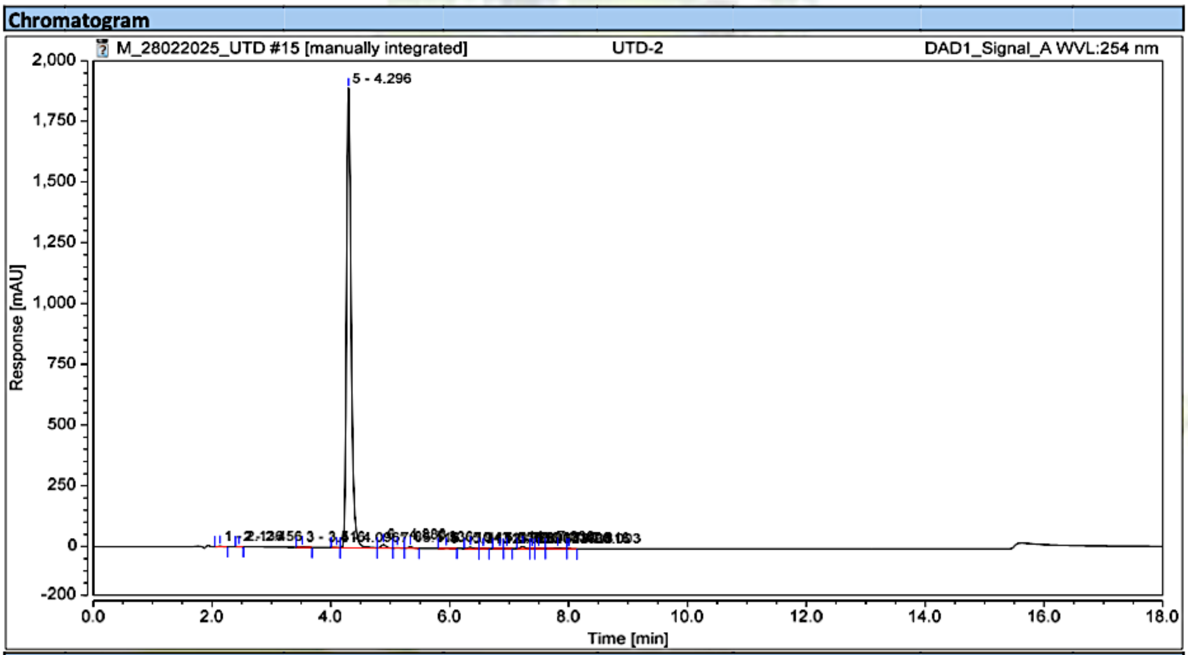

Integration Results

No.	Peak Name	Retention Time min	Area mAU*min	Height mAU	Relative Area %	Relative Height %	Amount n.a.
1		2.136	0.129	1.771	0.07	0.09	n.a.
2		2.456	0.009	0.135	0.01	0.01	n.a.
3		3.516	0.170	1.850	0.10	0.10	n.a.
4		4.096	0.010	0.177	0.01	0.01	n.a.
5		4.296	169.831	1890.510	97.63	97.67	n.a.
6		4.883	1.349	14.437	0.78	0.75	n.a.
7		5.116	0.137	1.579	0.08	0.08	n.a.
8		5.336	0.439	5.205	0.25	0.27	n.a.
9		5.943	0.153	1.557	0.09	0.08	n.a.
10		6.350	0.419	4.851	0.24	0.25	n.a.
11		6.563	0.040	0.408	0.02	0.02	n.a.
12		6.843	0.026	0.246	0.01	0.01	n.a.
13		6.963	0.038	0.520	0.02	0.03	n.a.
14		7.230	0.743	8.732	0.43	0.45	n.a.
15		7.383	0.031	0.452	0.02	0.02	n.a.
16		7.496	0.103	1.137	0.06	0.06	n.a.
17		7.816	0.306	1.735	0.18	0.09	n.a.
18		8.003	0.019	0.229	0.01	0.01	n.a.
**Total:**			**173.951**	**1935.531**	**100.00**	**100.00**	

Figure S8. HPLC chromatogram and peak description of **PyCN-2TA**.

S1.4 Synthesis and verification of Pm-2TA

4,6-di(thianthren-1-yl)pyrimidine (**Pm-2TA**) was synthesized in accordance with the general procedure from 4,6-dibromopyrimidine (0.20 g, 0.84 mmol), thianthrene-1-boronic acid (0.50 g, 1.92 mmol), tetrakis(triphenylphosphine)palladium(0) (0.05 g, 0.04 mmol) and potassium carbonate (0.70 g, 5.07 mmol). The pure product was isolated as a light green powder with 70 % yield (0.30 g, 0.59 mmol).

^{1}H NMR (400 MHz, CD_2Cl_2) δ 9.51 (d, *J* = 1.0 Hz, 1H, Pm), 7.88 (d, *J* = 1.1 Hz, 1H, Pm), 7.68 (dd, *J* = 7.7, 1.1 Hz, 2H, Ar), 7.63 (dd, *J* = 7.6, 1.1 Hz, 2H, Ar), 7.51 (dd, *J* = 7.6, 0.9 Hz, 2H, Ar), 7.41 (t, *J* = 7.6 Hz, 4H, Ar), 7.28 (td, *J* = 7.5, 1.3 Hz, 2H, Ar), 7.22 (td, *J* = 7.5, 1.3 Hz, 2H, Ar).
^{13}C NMR (101 MHz, CD_2Cl_2) δ 164.94, 157.57, 138.74, 136.50, 136.44, 136.36, 135.67, 130.65, 129.20, 128.83, 128.58, 128.06, 127.75, 127.32, 120.31.
HR-MS (m/z): calculated for $C_{28}H_{16}N_2S_4$ $[M]^+$ = 508.69, found $([M]+H)^+$ = 509.03

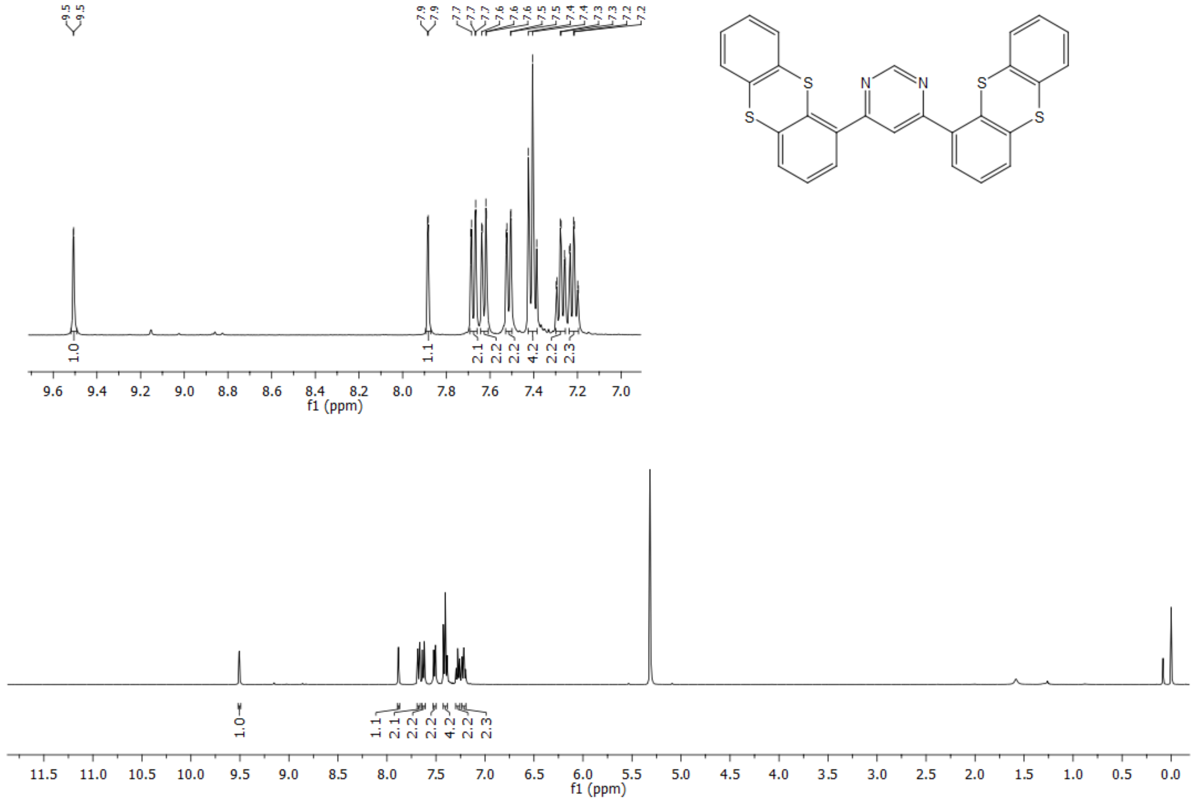

Figure S9. ^{1}H NMR spectrum of **Pm-2TA** in CD_2Cl_2.

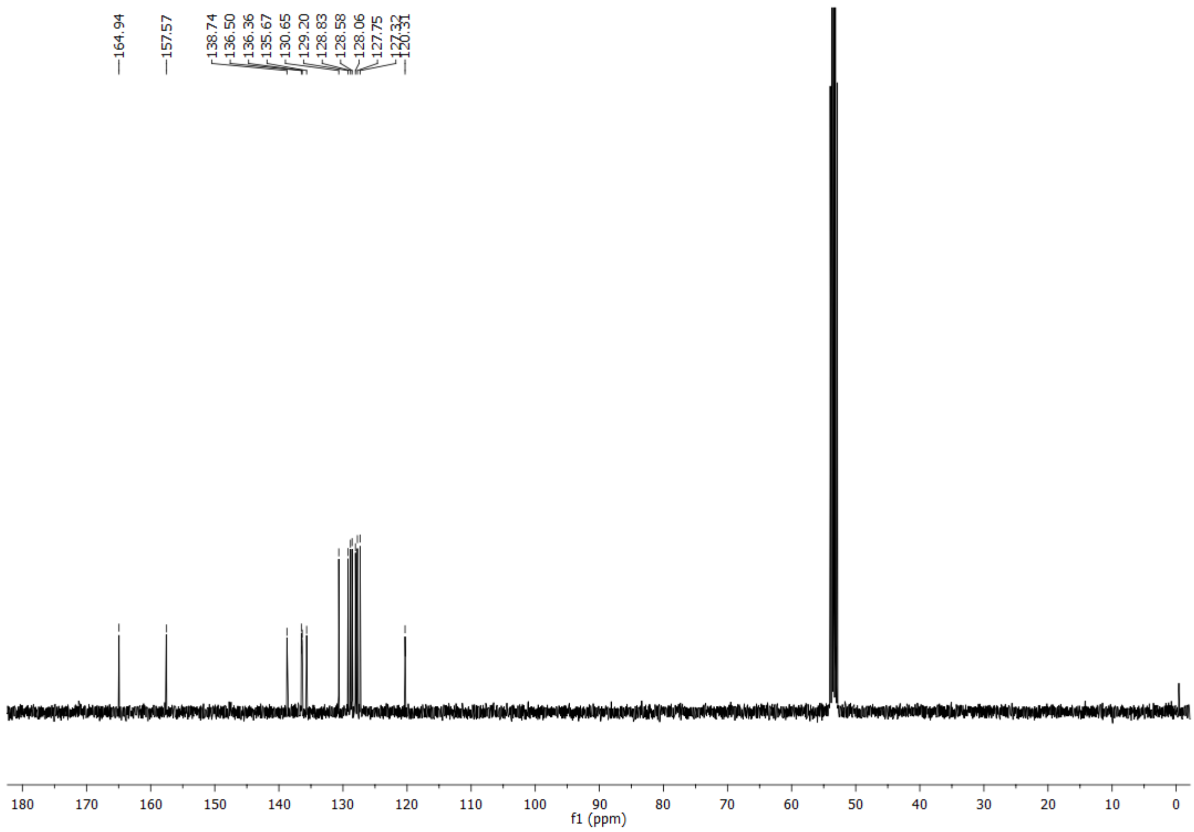

Figure S10. ^{13}C NMR spectrum of **Pm-2TA** in CD_2Cl_2.

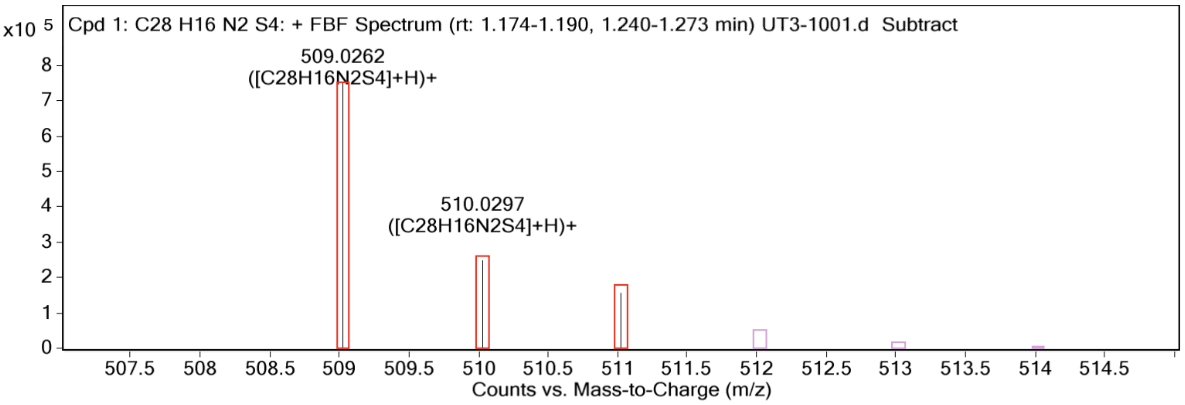

Figure S11. HRMS spectrum of **Pm-2TA**.

Chromatogram

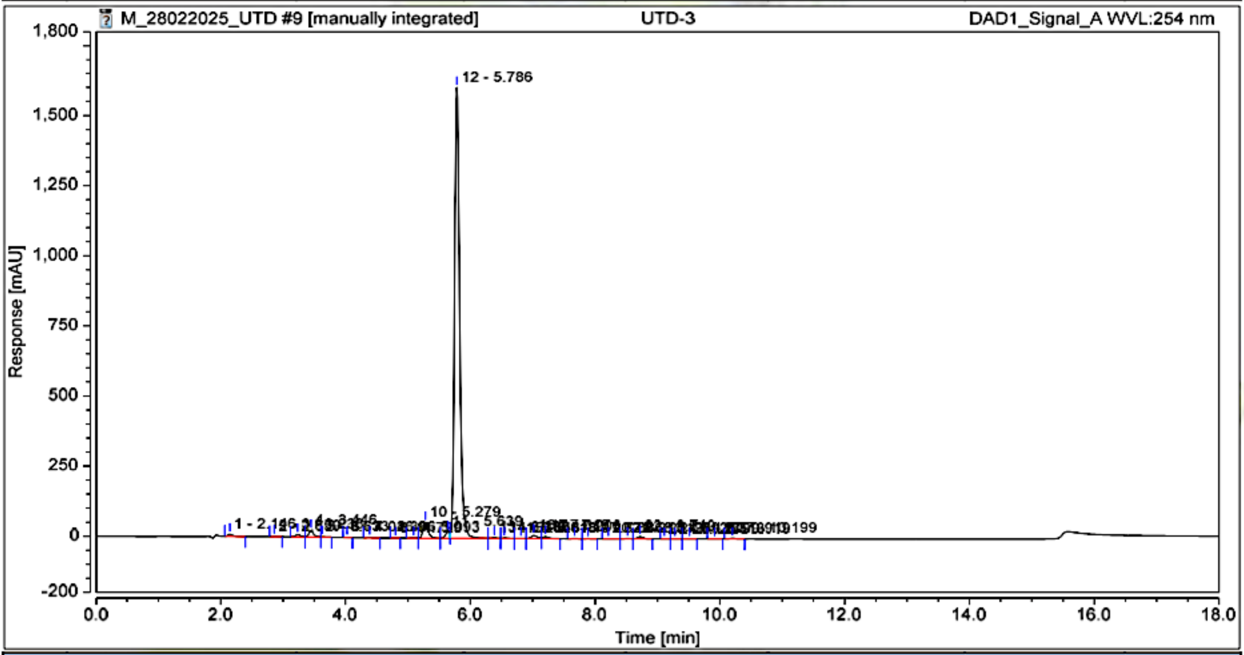

Integration Results

No.	Peak Name	Retention Time min	Area mAU*min	Height mAU	Relative Area %	Relative Height %	Amount n.a.
1		2.146	0.565	7.426	0.34	0.42	n.a.
2		2.859	0.069	0.830	0.04	0.05	n.a.
3		3.233	0.746	7.820	0.45	0.44	n.a.
4		3.446	2.034	22.807	1.24	1.29	n.a.
5		3.633	0.086	0.891	0.05	0.05	n.a.
6		4.026	0.019	0.238	0.01	0.01	n.a.
7		4.386	0.127	1.476	0.08	0.08	n.a.
8		4.799	0.016	0.238	0.01	0.01	n.a.
9		5.093	0.103	1.177	0.06	0.07	n.a.
10		5.279	5.150	55.487	3.13	3.14	n.a.
11		5.639	1.645	22.930	1.00	1.30	n.a.
12		5.786	149.980	1607.263	91.25	91.00	n.a.
13		6.386	0.230	2.719	0.14	0.15	n.a.
14		6.546	0.168	1.639	0.10	0.09	n.a.
15		6.813	0.051	0.603	0.03	0.03	n.a.
16		7.019	1.305	11.780	0.79	0.67	n.a.
17		7.206	0.700	7.051	0.43	0.40	n.a.
18		7.673	0.082	1.019	0.05	0.06	n.a.
19		7.886	0.033	0.370	0.02	0.02	n.a.
20		8.213	0.075	0.471	0.05	0.03	n.a.
21		8.519	0.102	1.095	0.06	0.06	n.a.
22		8.719	0.748	7.715	0.45	0.44	n.a.
23		9.113	0.011	0.145	0.01	0.01	n.a.
24		9.279	0.018	0.208	0.01	0.01	n.a.
25		9.513	0.026	0.231	0.02	0.01	n.a.
26		9.919	0.028	0.281	0.02	0.02	n.a.
27		10.199	0.255	2.377	0.15	0.13	n.a.
**Total:**			**164.370**	**1766.285**	**100.00**	**100.00**	

Figure S12. HPLC chromatogram and peak description of **Pm-2TA**.

S1.5 Synthesis and verification of PmCN-2TA

4,6-di(thianthren-1-yl)pyrimidine-5-carbonitrile (**PmCN-2TA**) was synthesized according to the general procedure from 4,6-dibromopyrimidine-5-carbonitrile (0.30 g, 1.14 mmol), thianthrene-1-boronic acid (0.68 g, 2.61 mmol), tetrakis(triphenylphosphine)palladium(0) (0.07 g, 0.06 mmol) and potassium carbonate (0.94 g, 6.81 mmol). The pure product was isolated as a dark green powder with 5.8 % yield (0.035 g, 0.06 mmol).

^{1}H NMR (500 MHz, CD_2Cl_2) δ 9.63 (s, *J* = 4.8 Hz, 1H, Pm), 7.79 (dd, *J* = 7.8, 1.2 Hz, 2H, Ar), 7.65 (dd, *J* = 7.6, 1.2 Hz, 2H, Ar), 7.60 – 7.58 (m, 2H, Ar), 7.54 – 7.47 (m, 4H, Ar), 7.36 – 7.33 (m, 2H, Ar), 7.30 – 7.26 (m, 2H, Ar).

^{13}C NMR (126 MHz, CD_2Cl_2) δ 168.68, 158.66, 137.76, 136.07, 136.04, 135.88, 134.76, 131.21, 129.30, 128.98, 128.66, 128.28, 127.92, 127.45.

HR-MS (m/z): calculated for $C_{29}H_{15}N_3S_4$ [M] = 533.70, found $([M]+H)^+$ = 534.02

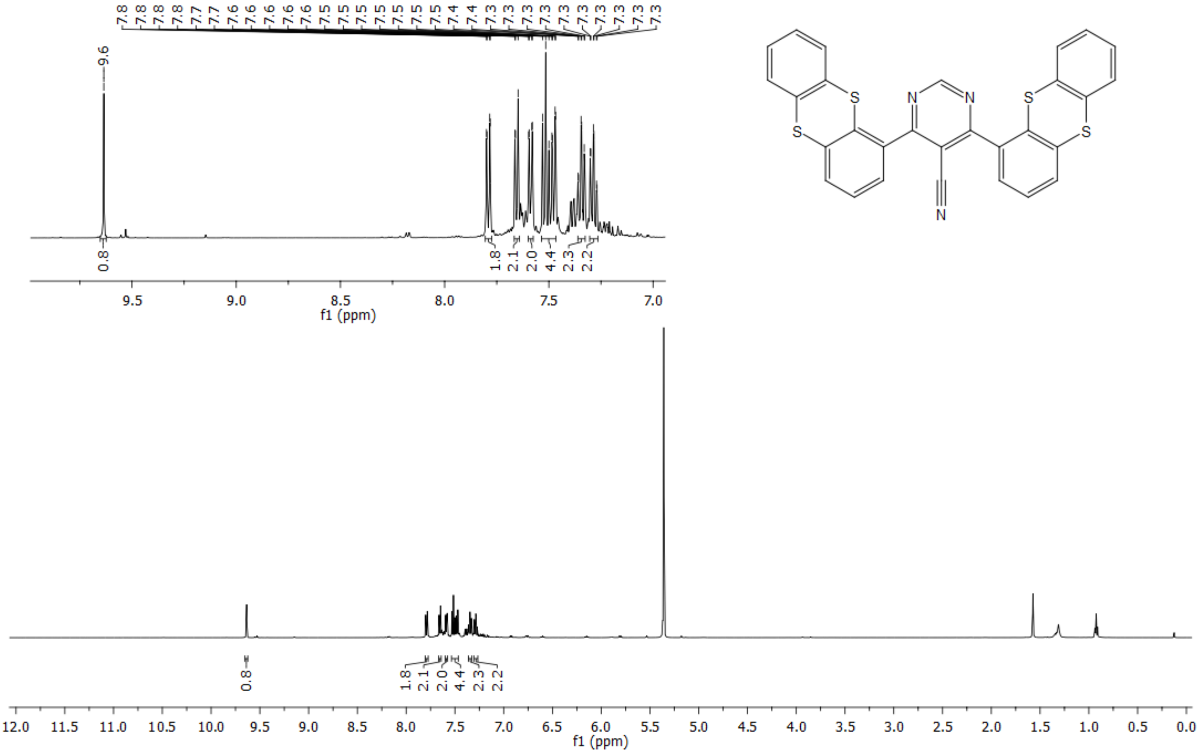

Figure S13. ^{1}H NMR spectrum of **PmCN-2TA** in CD_2Cl_2.

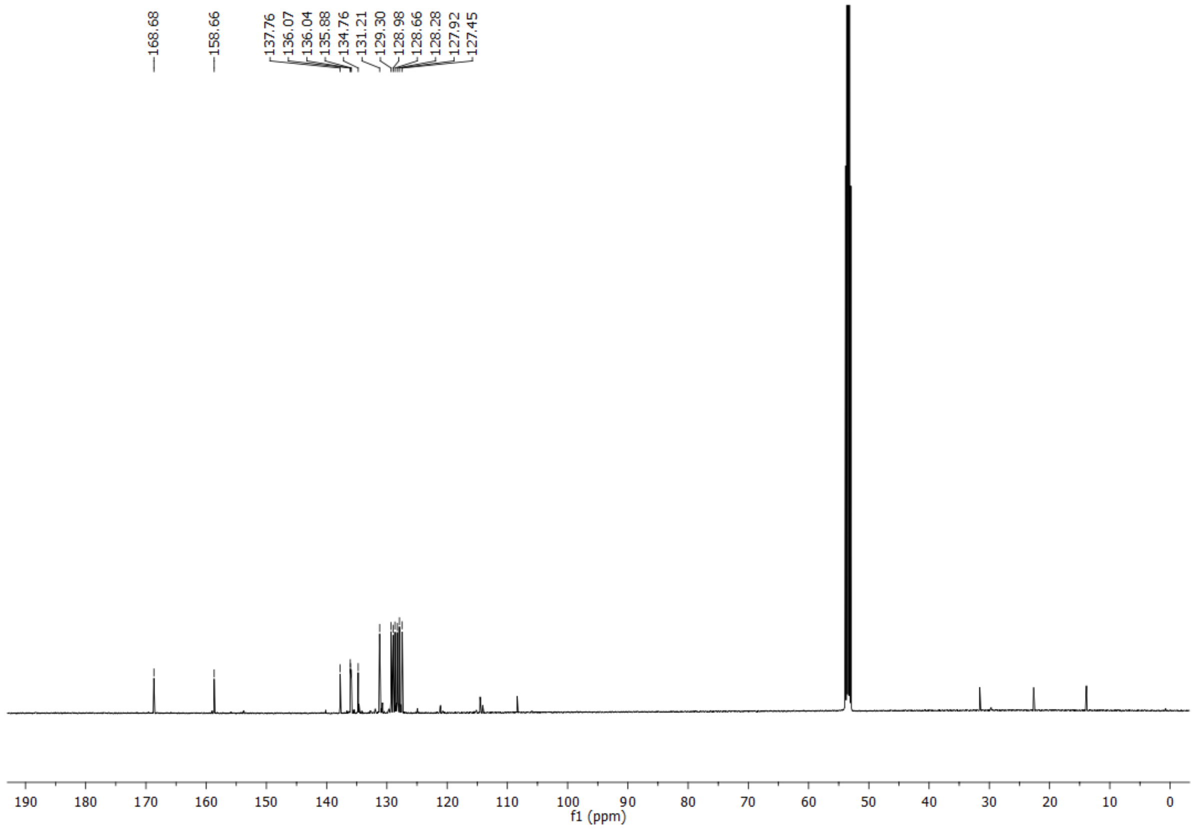

Figure S14. ^{13}C NMR spectrum of **PmCN-2TA** in CD_2Cl_2.

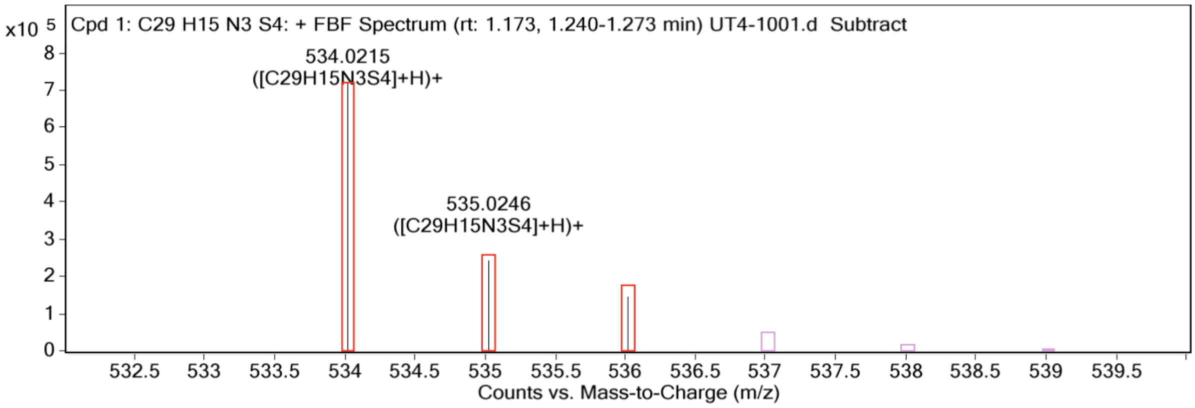

Figure S15. HRMS spectrum of **PmCN-2TA**.

Chromatogram

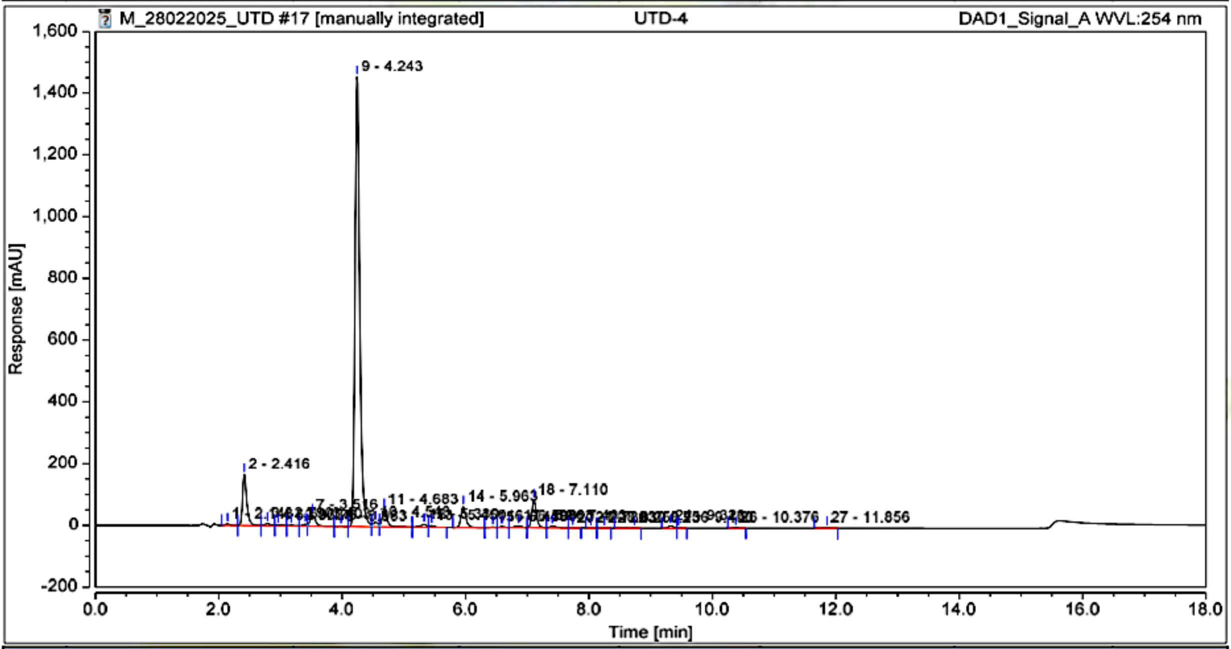

Integration Results

No.	Peak Name	Retention Time min	Area mAU*min	Height mAU	Relative Area %	Relative Height %	Amount n.a.
1		2.143	0.398	4.982	0.23	0.25	n.a.
2		2.416	12.935	165.141	7.43	8.43	n.a.
3		2.790	0.830	7.270	0.48	0.37	n.a.
4		2.963	0.340	2.174	0.20	0.11	n.a.
5		3.176	0.278	1.813	0.16	0.09	n.a.
6		3.403	0.518	6.634	0.30	0.34	n.a.
7		3.516	3.677	36.960	2.11	1.89	n.a.
8		3.983	0.098	0.840	0.06	0.04	n.a.
9		4.243	129.270	1456.769	74.21	74.34	n.a.
10		4.543	1.396	15.160	0.80	0.77	n.a.
11		4.683	5.337	56.172	3.06	2.87	n.a.
12		5.330	1.104	9.472	0.63	0.48	n.a.
13		5.450	0.553	5.909	0.32	0.30	n.a.
14		5.963	6.145	67.604	3.53	3.45	n.a.
15		6.443	0.139	1.662	0.08	0.08	n.a.
16		6.590	0.378	4.157	0.22	0.21	n.a.
17		6.863	0.838	6.255	0.48	0.32	n.a.
18		7.110	7.603	89.119	4.36	4.55	n.a.
19		7.403	0.739	5.578	0.42	0.28	n.a.
20		7.736	0.080	0.788	0.05	0.04	n.a.
21		8.030	0.072	0.963	0.04	0.05	n.a.
22		8.250	0.051	0.414	0.03	0.02	n.a.
23		8.736	0.120	0.776	0.07	0.04	n.a.
24		9.323	0.724	7.864	0.42	0.40	n.a.
25		9.450	0.105	1.261	0.06	0.06	n.a.
26		10.376	0.229	2.168	0.13	0.11	n.a.
27		11.856	0.234	1.691	0.13	0.09	n.a.
**Total:**			**174.191**	**1959.599**	**100.00**	**100.00**	

Figure S16. HPLC chromatogram and peak description of **PmCN-2TA**.

S2 Crystallographic details

For **BP-2TA**, **Py-2TA**, **PyCN-2TA, and Pm-2TA** single crystals were obtained by slow diffusion of *n*-hexane into an ethylacetate solution of the compounds, allowing for X-ray diffraction analyses. For **PmCN-2TA**, no crystals were obtained suitable for X-ray analysis.

**Compound**	**BP-2TA**	**Py-2TA**	**PyCN-2TA**	**Pm-2TA x CH_2Cl_2**
Empirical formula	$C_{37}H_{22}OS_4$	$C_{29}H_{17}NS_4$	$C_{30}H_{16}N_2S_4$	$C_{29}H_{18}Cl_2N_2S_4$
Formula weight	610.78	507.67	532.69	593.59
Temperature/K	99.98(10)	100.01(10)	100.00(10)	100.01(10)
Crystal system	Monoclinic	monoclinic	Monoclinic	triclinic
Space group	$P2_1/n$	I2/a	$P2_1/n$	P-1
a/Å	10.63440(10)	15.13566(11)	7.77190(10)	7.4913(2)
b/Å	20.9375(2)	6.95148(4)	22.1140(3)	11.1972(3)
c/Å	13.40470(10)	21.64403(15)	14.2893(2)	16.8013(5)
α/°	90	90	90	106.090(2)
β/°	102.9160(10)	90.0748(6)	95.2470(10)	93.322(2)
γ/°	90	90	90	106.258(2)
Volume/Å^3	2909.14(5)	2277.28(3)	2445.58(6)	1285.85(6)
Z	4	4	4	2
ρ_{calc} *g/cm^3*	1.395	1.481	1.447	1.533
μ/mm^{-1}	3.235	3.982	0.413	5.494
F(000)	1264.0	1048.0	1096.0	608.0
Crystal size/mm^3	0.18 × 0.12 × 0.1	0.22 × 0.12 × 0.03	0.22 × 0.16 × 0.07	0.23 × 0.13 × 0.09
Radiation	Cu K_α (λ = 1.54184)	Cu K_α (λ = 1.54184)	Mo K_α (λ = 0.71073)	Cu K_α (λ = 1.54184)
2Θ range for data collection/°	7.976 to 153.462	8.17 to 153.372	4.666 to 59.52	5.534 to 153.614
Index ranges	$-13 \le h \le 13$, $-26 \le k \le 26$, $-16 \le l \le 12$	$-19 \le h \le 18$, $-8 \le k \le 7$, $-27 \le l \le 27$	$-10 \le h \le 10$, $-30 \le k \le 30$, $-19 \le l \le 19$	$-8 \le h \le 9$, $-9 \le k \le 14$, $-20 \le l \le 20$
Reflections collected	21613	12256	25066	14015
Independent reflections	6081 [R_{int} = 0.0330, R_{sigma} = 0.0288]	2388 [R_{int} = 0.0429, R_{sigma} = 0.0252]	6233 [R_{int} = 0.0262, R_{sigma} = 0.0291]	5349 [R_{int} = 0.0222, R_{sigma} = 0.0267]
Data/restraints/parameters	6081/0/379	2388/0/155	6233/0/325	5349/0/334
Goodness-of-fit on F^2	1.024	1.057	1.022	1.045
Final R indexes [I>=2σ (I)]	R_1 = 0.0337, wR_2 = 0.0862	R_1 = 0.0346, wR_2 = 0.0939	R_1 = 0.0338, wR_2 = 0.0759	R_1 = 0.0295, wR_2 = 0.0739
Final R indexes [all data]	R_1 = 0.0382, wR_2 = 0.0899	R_1 = 0.0357, wR_2 = 0.0953	R_1 = 0.0447, wR_2 = 0.0810	R_1 = 0.0319, wR_2 = 0.0755
Largest diff. peak/ hole / e Å^{-3}	0.33/-0.34	0.34/-0.52	0.40/-0.27	0.67/-0.71
CCDC Number	2388221	2388220	2388219	2388222

S3 Thermal properties

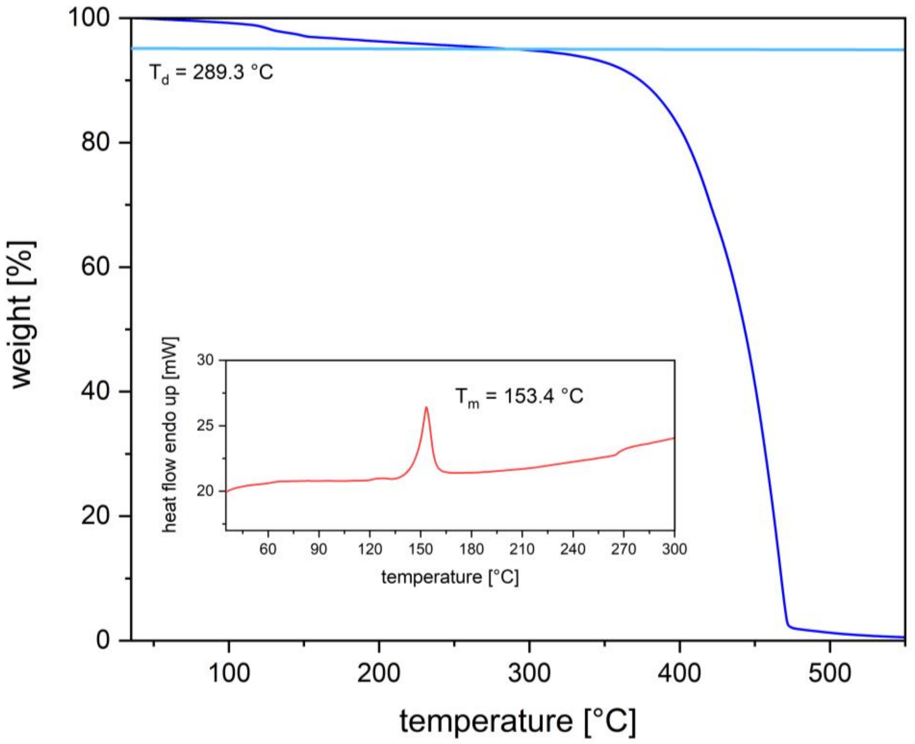

Figure S17. TGA and DSC (insert) results from simultaneous thermal analysis of **Py-2TA** (T_d was obtained at 5 % weight loss).

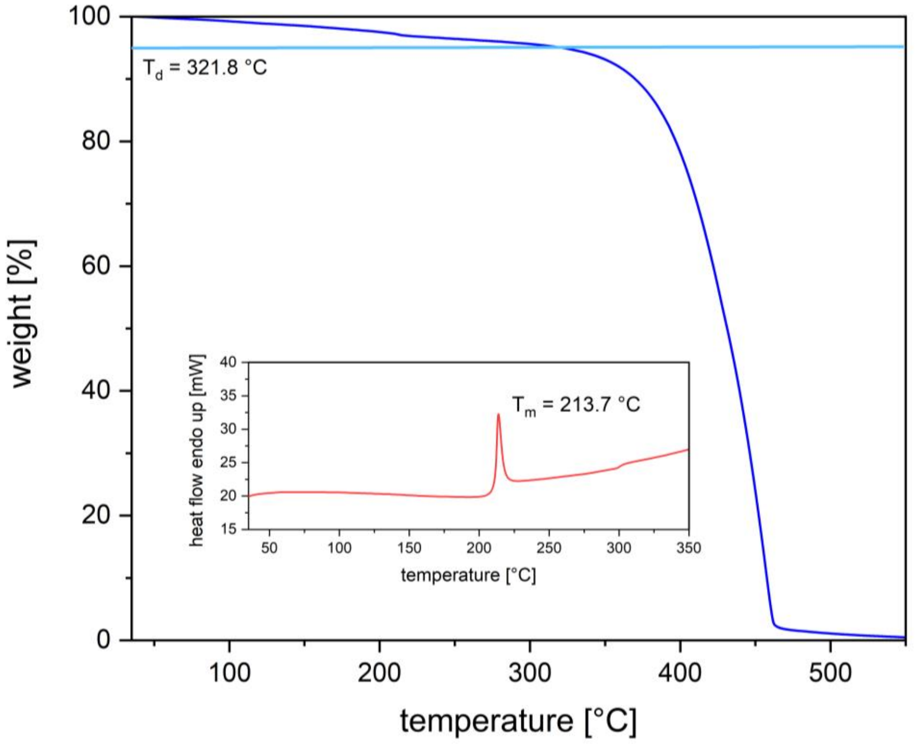

Figure S18. TGA and DSC (insert) results from simultaneous thermal analysis of **PyCN-2TA** (T_d was obtained at 5 % weight loss).

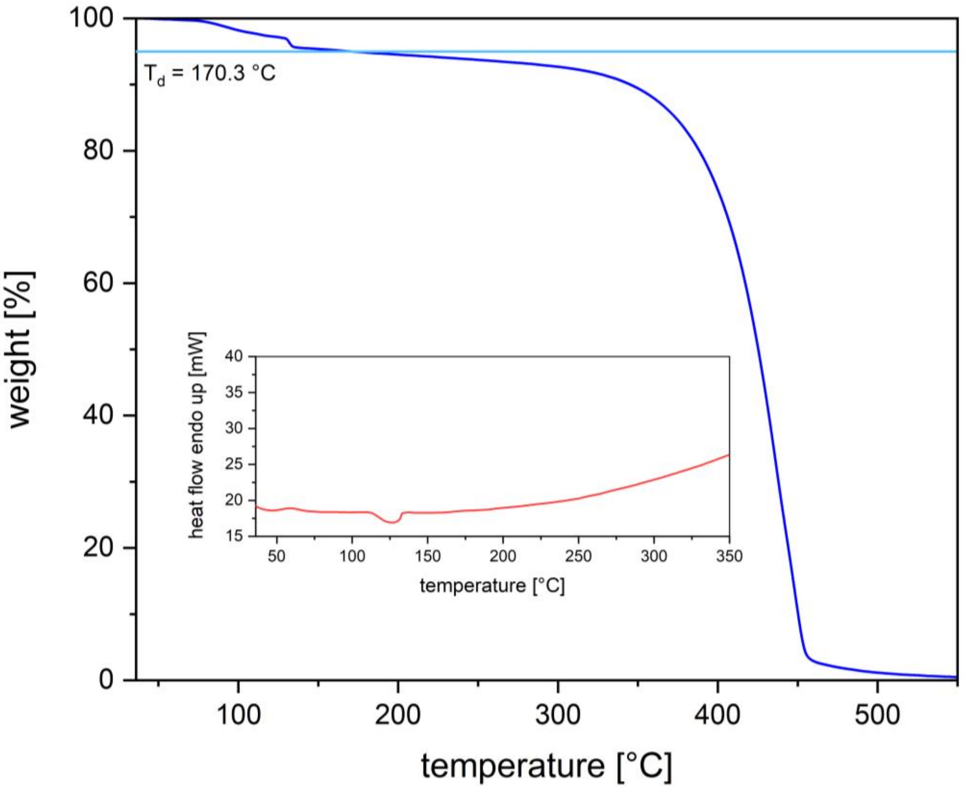

Figure S19. TGA and DSC (insert) results from simultaneous thermal analysis of **Pm-2TA** (T_d was obtained at 5 % weight loss).

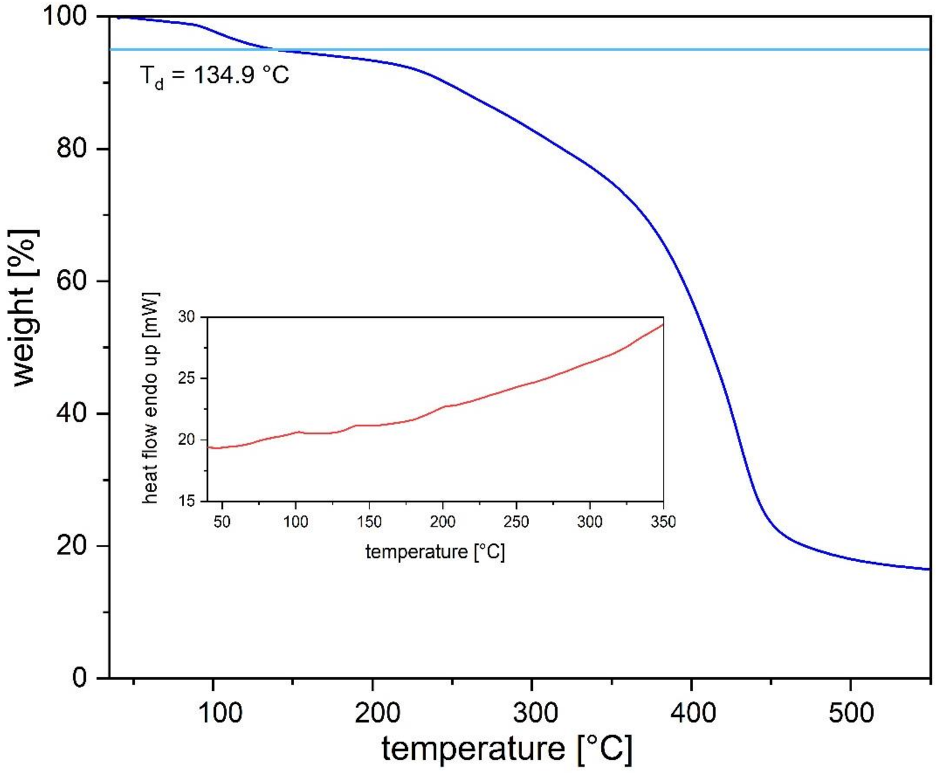

Figure S20. TGA and DSC (insert) results from simultaneous thermal analysis of **PmCN-2TA** (T_d was obtained at 5% weight loss).

S4 Further results from materials simulations

This section contains a detailed visual characterization of the electronic structure of the RTP emitter molecules and their donor and acceptor moieties based on quantum chemical simulations at the CAM-B3LYP/6-311G** level of theory.

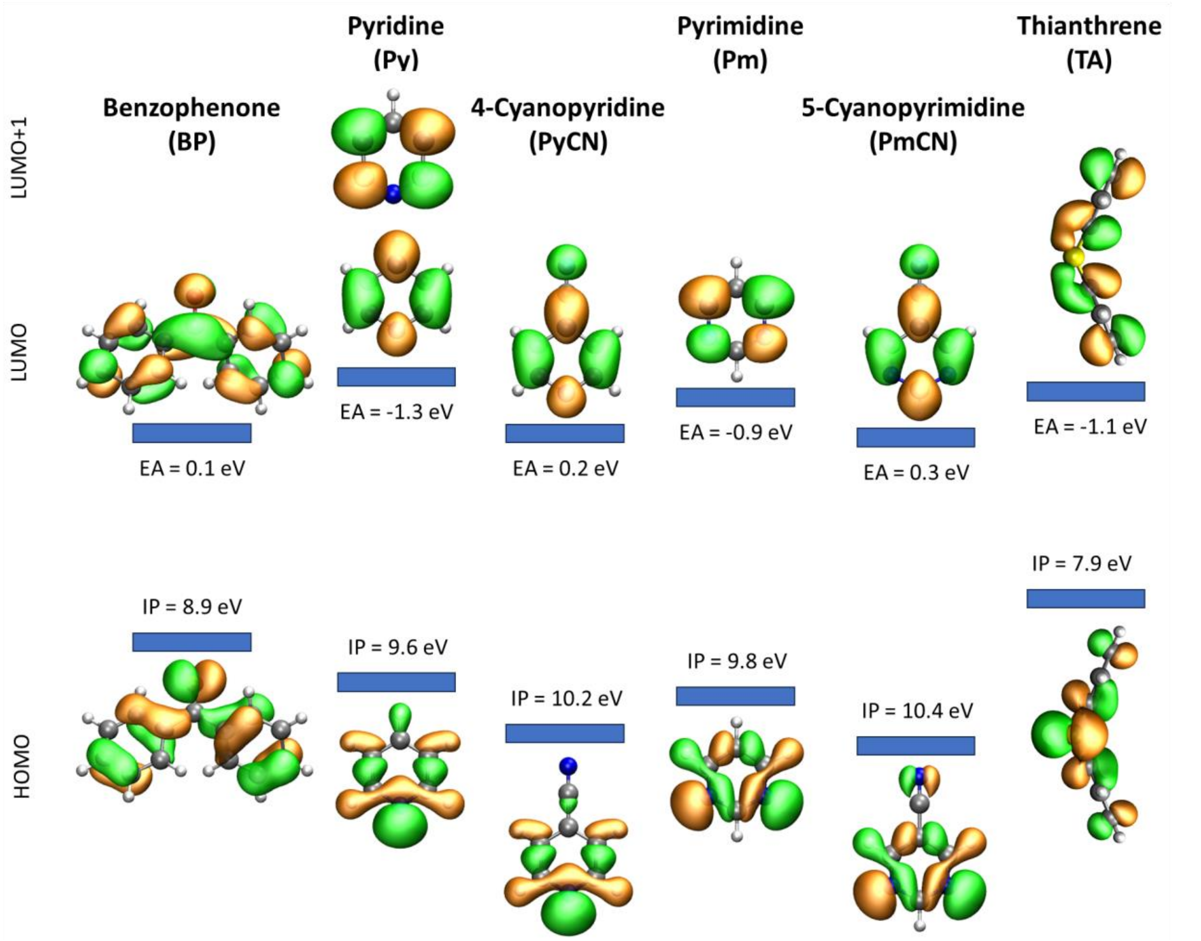

Figure S21. Frontier molecular orbitals and corresponding energy levels in gas phase for the acceptor and donor moieties of the analyzed RTP emitter materials.

Table S1. Transitions to excited states of **BP-2TA** from its relaxed ground state S_0 (CAM-B3LYP/6-311G**). Six further excited triplet states exist between T_1 and S_1. Wavelengths in PS are estimated by an empirical red-shift of 500 meV. The natural transition orbitals (NTOs) of most relevant transitions are depicted in Fig. S22.

Final state	Wavelength (estimated wavelength in PS film)	Osc. strength	Character of transition according to NTOs
T_1	407 nm (488 nm)	-	BP → BP
S_1	319 nm (367 nm)	0.001	BP → BP
S_2	272 nm (305 nm)	0.011	TA → TA
S_3	272 nm (305 nm)	0.000	TA → TA
S_4	267 nm (299 nm)	0.451	hybrid → hybrid

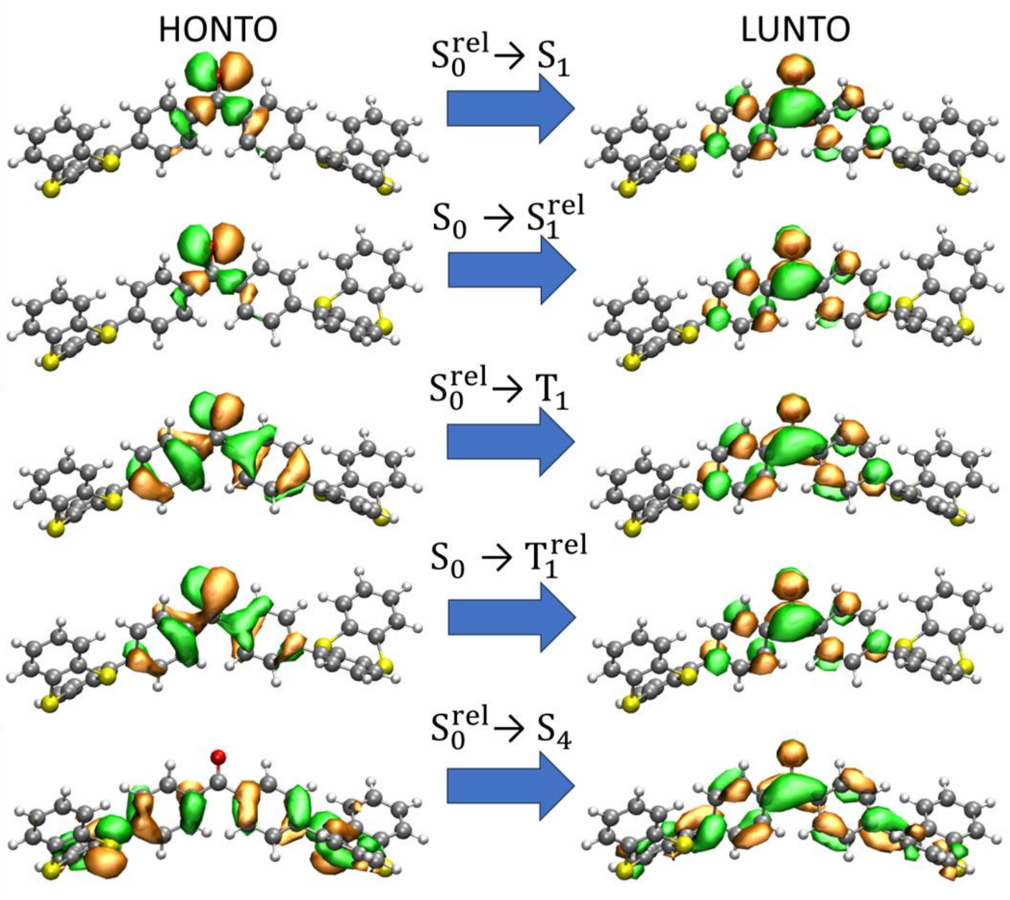

Figure S22. Natural transition orbitals (NTOs) with dominant contributions for relevant excitations of **BP-2TA** for different relaxed geometries.

Table S2. Transitions to excited states of **Py-2TA** from its relaxed ground state S_0 (CAM-B3LYP/6-311G**). 14 further excited triplet states exist between T_1 and S_1. Wavelengths in PS are estimated by an empirical red-shift of 500 meV. The natural transition orbitals (NTOs) of most relevant transitions are depicted in Fig. S23.

Final state	Wavelength (estimated wavelength in PS film)	Osc. strength	Character of transition according to NTOs
T_1	381 nm (450 nm)	-	hybrid → hybrid
S_1	270 nm (302 nm)	0.000	TA → TA
S_2	270 nm (302 nm)	0.001	TA → TA
S_3	258 nm (288 nm)	0.003	hybrid → hybrid
S_4	257 nm (287 nm)	0.005	TA → hybrid
S_5	249 nm (277 nm)	0.028	Py → Py
S_6	248 nm (275 nm)	0.007	TA → TA
S_7	247 nm (275 nm)	0.009	TA → TA
S_8	243 nm (269 nm)	0.017	hybrid → hybrid
S_9	239 nm (265 nm)	0.452	TA → hybrid

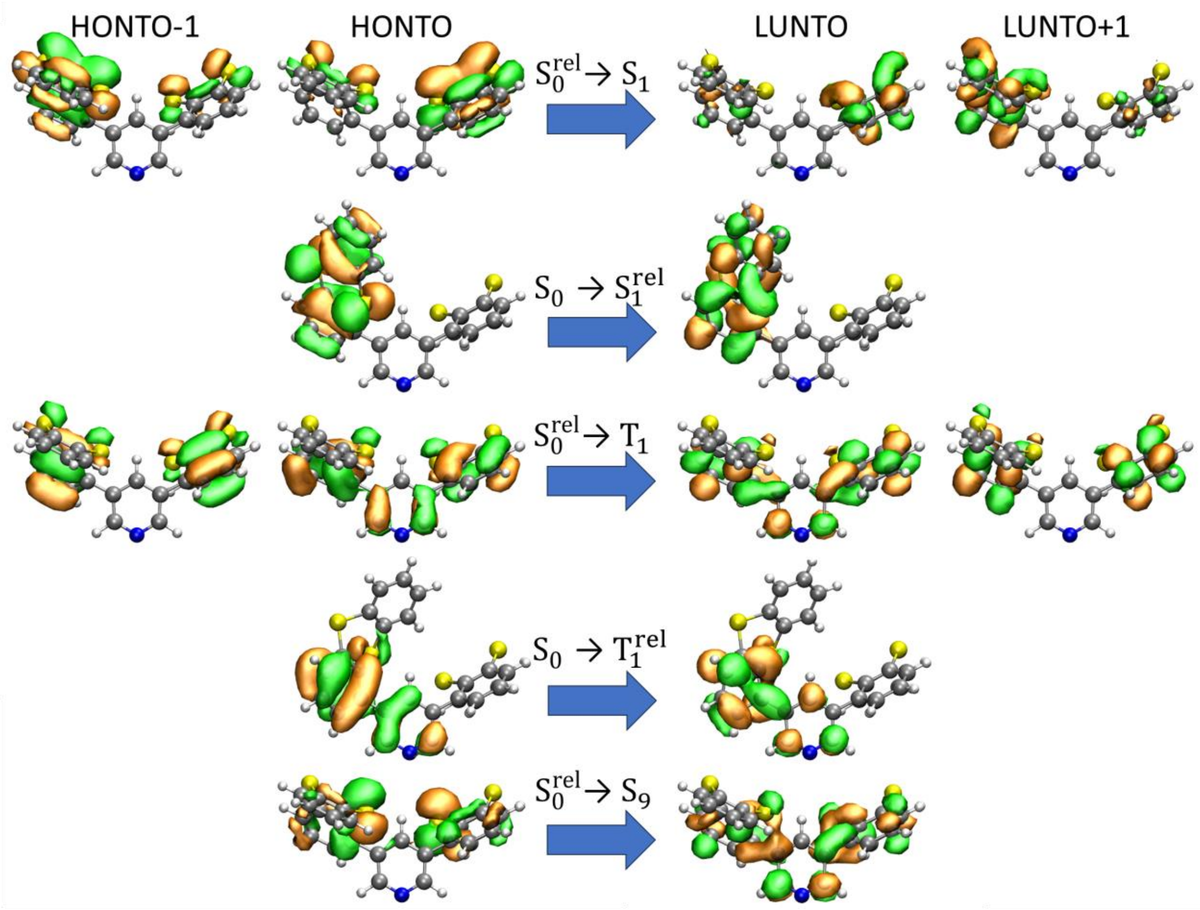

Figure S23. Natural transition orbitals (NTOs) with dominant contributions for relevant excitations of **Py-2TA** for different relaxed geometries.

Table S3. Transitions to excited states of **PyCN-2TA** from its relaxed ground state S_0 (CAM-B3LYP/6-311G**). Nine further excited triplet states exist between T_1 and S_1. Wavelengths in PS are estimated by an empirical red-shift of 500 meV. The natural transition orbitals (NTOs) of most relevant transitions are depicted in Fig. S24.

Final state	Wavelength (estimated wavelength in PS film)	Osc. strength	Character of transition according to NTOs
T_1	383 nm (454 nm)	-	hybrid → hybrid
S_1	286 nm (324 nm)	0.024	hybrid → PyCN
S_2	283 nm (320 nm)	0.000	TA → PyCN
S_3	271 nm (305 nm)	0.002	hybrid → hybrid
S_4	269 nm (302 nm)	0.000	TA → TA
S_5	264 nm (296 nm)	0.016	hybrid → PyCN
S_6	253 nm (282 nm)	0.053	hybrid → PyCN
S_7	253 nm (282 nm)	0.009	TA → hybrid
S_8	247 nm (275 nm)	0.027	hybrid → PyCN
S_9	247 nm (274 nm)	0.017	TA → PyCN
S_{10}	246 nm (273 nm)	0.049	TA → TA
S_{11}	244 nm (271 nm)	0.001	TA → hybrid
S_{12}	238 nm (263 nm)	0.113	hybrid → hybrid

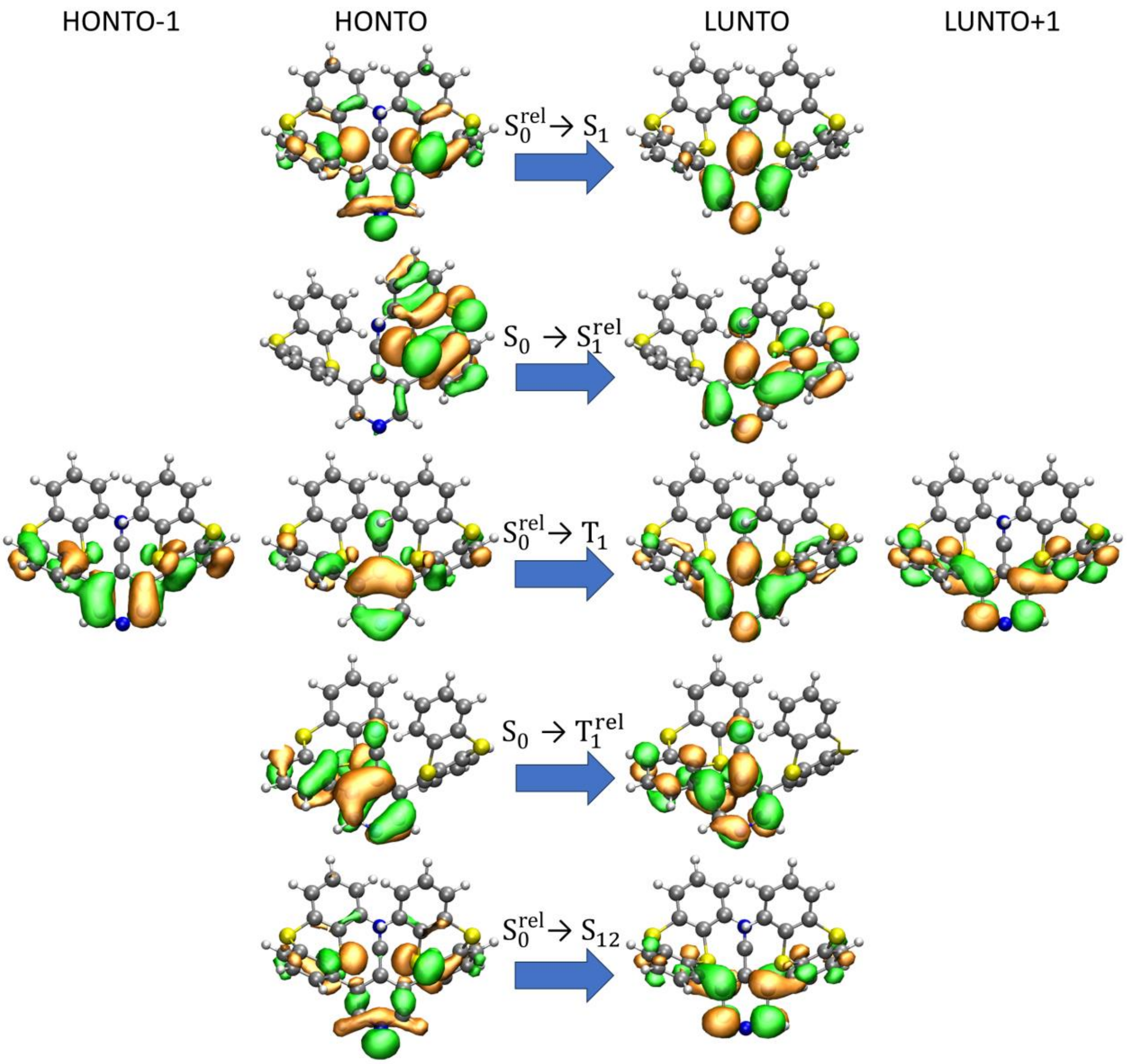

Figure S24. Natural transition orbitals (NTOs) with dominant contributions for relevant excitations of **PyCN-2TA** for different relaxed geometries.

Table S4. Transitions to excited states of **Pm-2TA** from its relaxed ground state S_0 (CAM-B3LYP/6-311G**). Eight further excited triplet states exist between T_1 and S_1. Wavelengths in PS are estimated by an empirical red-shift of 500 meV. The natural transition orbitals (NTOs) of most relevant transitions are depicted in Fig. S25.

Final state	Wavelength (estimated wavelength in PS film)	Osc. strength	Character of transition according to NTOs
T_1	393 nm (466 nm)	-	hybrid → hybrid
S_1	292 nm (330 nm)	0.137	hybrid → hybrid
S_2	287 nm (324 nm)	0.012	TA → hybrid
S_3	277 nm (311 nm)	0.023	Pm → Pm
S_4	269 nm (301 nm)	0.001	TA → TA
S_5	268 nm (300 nm)	0.016	TA → TA
S_6	259 nm (290 nm)	0.370	hybrid → hybrid

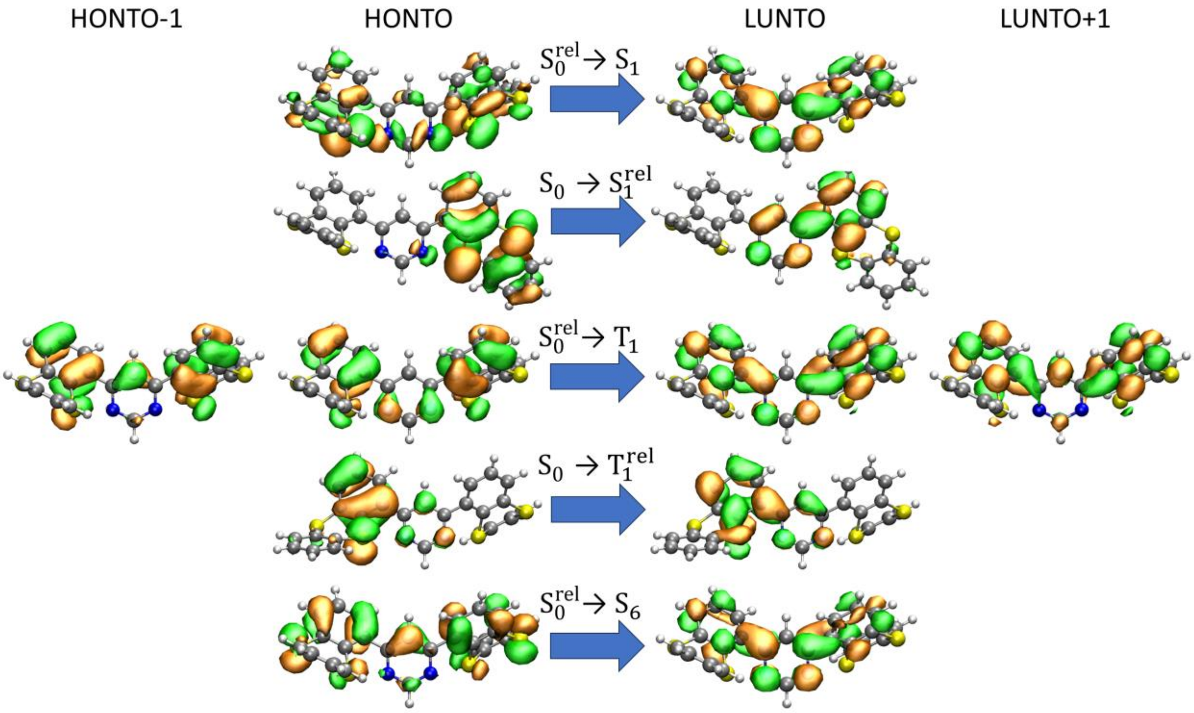

Figure S25. Natural transition orbitals (NTOs) with dominant contributions for relevant excitations of **Pm-2TA** for different relaxed geometries.

Table S5. Transitions to excited states of **PmCN-2TA** from its relaxed ground state S_0 (CAM-B3LYP/6-311G**). Eight further excited triplet states exist between T_1 and S_1. Wavelengths in PS are estimated by an empirical red-shift of 500 meV. The natural transition orbitals (NTOs) of most relevant transitions are depicted in Fig. S26.

Final state	Wavelength (estimated wavelength in PS film)	Osc. strength	Character of transition according to NTOs
T_1	394 nm (469 nm)	-	hybrid → hybrid
S_1	302 nm (344 nm)	0.073	TA → hybrid
S_2	301 nm (343 nm)	0.008	TA → PmCN
S_3	281 nm (317 nm)	0.000	PmCN → PmCN
S_4	278 nm (313 nm)	0.013	PmCN → PmCN
S_5	270 nm (303 nm)	0.000	hybrid → PmCN
S_6	269 nm (302 nm)	0.036	TA → hybrid
S_7	267 nm (296 nm)	0.251	hybrid → hybrid

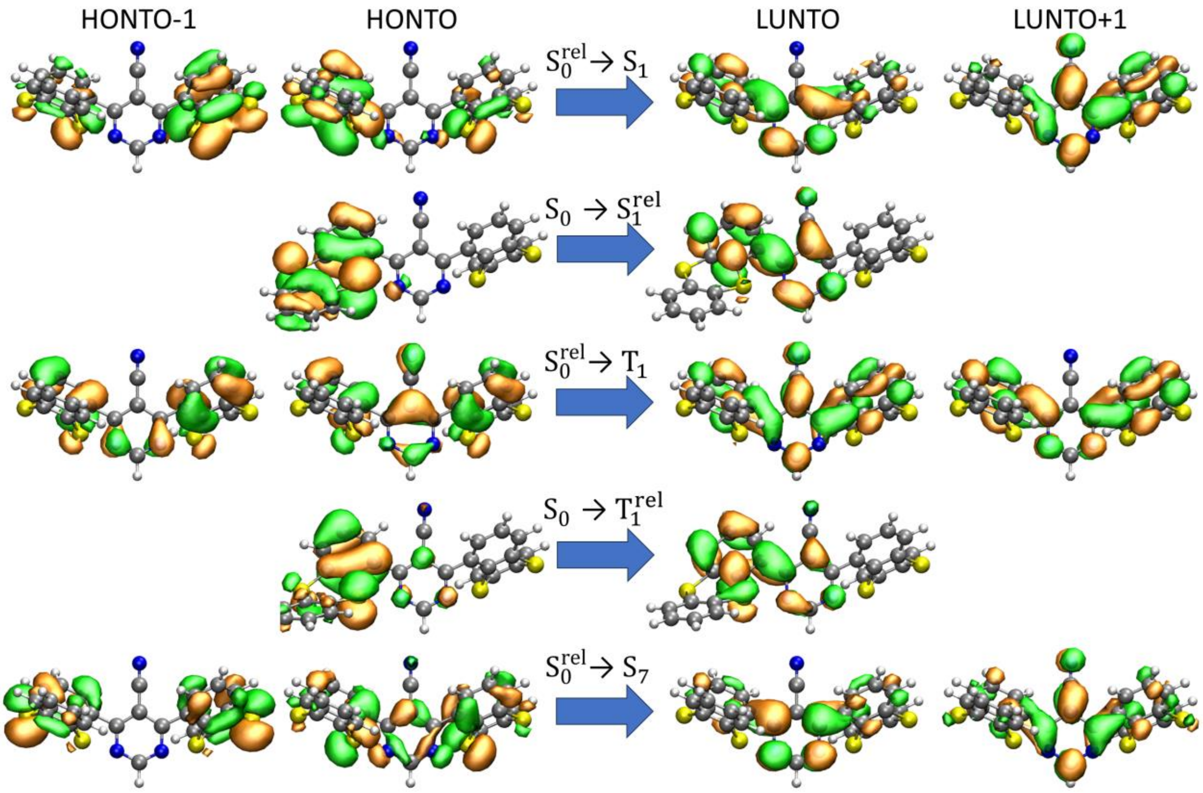

Figure S26. Natural transition orbitals (NTOs) with dominant contributions for relevant excitations of **PmCN-2TA** for different relaxed geometries.

S5 Molecular geometries

In the following subsections, the relaxed geometries of **BP-2TA**, **Py-2TA**, **PyCN-2TA**, **Pm-2TA**, and **PmCN-2TA** in their S_0, S_1, and T_1 state are given based on quantum chemical simulations at the CAM-B3LYP/6-311G** level of theory. These geometries represent global energy minima. However, all molecules exhibit a large variation of their three-dimensional conformation, including local minima, that is energetically accessible even at room temperature.

S5.1 BP-2TA

S5.1.1 S_0

C	1.190226	-1.033579	0.325006
C	1.230423	-0.413750	1.570067
C	2.462704	-0.244915	2.201886
C	3.630185	-0.648996	1.585954
C	3.594325	-1.266002	0.334264
C	2.362093	-1.464681	-0.280391
H	0.241984	-1.204330	-0.168016
C	0.000088	0.000975	2.314364
H	2.479778	0.202864	3.187199
H	4.582951	-0.500796	2.078641
H	2.320913	-1.954746	-1.245849
O	0.000159	0.001435	3.526925
C	-3.594481	1.266438	0.333872
C	-3.630191	0.649789	1.585741
C	-2.462611	0.246184	2.201806
C	-1.230378	0.415115	1.569937
C	-1.190321	1.034589	0.324696
C	-2.362287	1.465253	-0.280822
H	-4.582918	0.501508	2.078477
H	-2.479611	-0.201283	3.187262
H	-0.242116	1.205400	-0.168379
H	-2.321215	1.955053	-1.246420
C	7.164601	-2.828857	-1.448082
C	6.131730	-3.668440	-1.069045
C	4.982045	-3.139403	-0.506213
C	4.841026	-1.767357	-0.308026
H	8.078727	-3.230578	-1.866738
H	6.230416	-4.739280	-1.196739
H	4.182120	-3.795731	-0.186185
C	5.870289	-0.916402	-0.732342
C	7.025645	-1.454064	-1.303082
S	8.317016	-0.403323	-1.927585
S	5.670347	0.852267	-0.642564
C	8.432210	0.791772	-0.619567
C	7.278173	1.336752	-0.061394
C	7.381874	2.301233	0.935414

C	8.628764	2.747375	1.344127
C	9.778239	2.203604	0.787540
C	9.681203	1.214116	-0.177972
H	6.480672	2.704060	1.380906
H	8.701740	3.510745	2.108809
H	10.754157	2.539623	1.115424
H	10.573434	0.767671	-0.599094
C	-7.165046	2.828097	-1.448956
C	-6.132295	3.667967	-1.070231
C	-4.982519	3.139298	-0.507244
C	-4.841287	1.767343	-0.308565
H	-8.079236	3.229532	-1.867745
H	-6.231142	4.738747	-1.198299
H	-4.182692	3.795862	-0.187455
C	-5.870429	0.916083	-0.732577
C	-7.025873	1.453376	-1.303488
S	-8.317082	0.402227	-1.927646
S	-5.670199	-0.852533	-0.642307
C	-8.432064	-0.792474	-0.619250
C	-7.277928	-1.337101	-0.060941
C	-7.381449	-2.301296	0.936163
C	-8.628258	-2.747508	1.345046
C	-9.777833	-2.204090	0.788322
C	-9.680980	-1.214882	-0.177495
H	-6.480170	-2.703848	1.381749
H	-8.701093	-3.510658	2.109962
H	-10.753690	-2.540164	1.116333
H	-10.573292	-0.768711	-0.598734

S5.1.2 S_1

C	1.336710	-0.115888	-0.206419
C	0.750034	1.158743	-0.159875
C	-0.647980	1.253224	-0.285817
C	-1.422513	0.117883	-0.413863
C	-0.842569	-1.149532	-0.432574
C	0.547425	-1.241763	-0.334209
H	2.412176	-0.220320	-0.173273
C	1.537583	2.371391	-0.050242
H	-1.119919	2.226590	-0.261230
H	-2.497464	0.212469	-0.503551
H	1.017419	-2.217931	-0.364149
O	0.979889	3.458786	-0.503308
C	5.447258	2.912535	1.571602
C	4.915533	3.811640	0.648612
C	3.651736	3.629465	0.124261
C	2.870196	2.522214	0.501548
C	3.394353	1.631887	1.452091
C	4.660854	1.828085	1.966306
H	5.503019	4.667399	0.340331

H	3.263284	4.332850	-0.600393
H	2.796678	0.804899	1.809169
H	5.050926	1.128074	2.696049
C	-3.181353	-4.672384	-1.135907
C	-2.175359	-4.297873	-2.009437
C	-1.426264	-3.161430	-1.754431
C	-1.663216	-2.374698	-0.627908
H	-3.791881	-5.543998	-1.335382
H	-1.987497	-4.883070	-2.901129
H	-0.656912	-2.851508	-2.450954
C	-2.653065	-2.782761	0.277744
C	-3.404029	-3.930855	0.017713
S	-4.609298	-4.526013	1.181992
S	-2.885596	-1.906822	1.813080
C	-5.412742	-3.008669	1.632952
C	-4.659477	-1.869575	1.907699
C	-5.297982	-0.697950	2.300743
C	-6.674980	-0.674612	2.456237
C	-7.424691	-1.810031	2.181373
C	-6.797556	-2.968912	1.751955
H	-4.708112	0.189991	2.491975
H	-7.163559	0.236357	2.779152
H	-8.502272	-1.790687	2.287722
H	-7.377684	-3.851450	1.512509
C	9.237537	3.609535	3.448556
C	8.073277	3.546156	4.194090
C	6.862299	3.308775	3.565708
C	6.784645	3.131355	2.184900
H	10.187962	3.819616	3.922693
H	8.107749	3.701072	5.265386
H	5.946675	3.286145	4.143826
C	7.972196	3.151242	1.439331
C	9.191168	3.388445	2.077695
S	10.725583	3.338714	1.180169
S	7.949400	2.767755	-0.301665
C	10.284542	4.244055	-0.281723
C	9.074901	3.993024	-0.925294
C	8.759016	4.679041	-2.093400
C	9.661041	5.580347	-2.636393
C	10.865782	5.830336	-1.993820
C	11.168972	5.178553	-0.808928
H	7.807545	4.494771	-2.576829
H	9.415590	6.098097	-3.555379
H	11.566342	6.544996	-2.407753
H	12.096076	5.386020	-0.289130

S5.1.3 T_1

C	1.295932	-0.945787	-0.105375
C	1.243220	-0.388319	1.184707
C	2.448915	-0.275824	1.906960
C	3.646717	-0.664689	1.346298
C	3.700378	-1.189936	0.053563
C	2.503707	-1.327038	-0.654401
H	0.382472	-1.099146	-0.663915
C	0.000072	0.001196	1.800089
H	2.429805	0.142299	2.905083
H	4.561918	-0.560468	1.915542
H	2.524774	-1.746575	-1.653514
O	0.000063	0.001944	3.127871
C	-3.700637	1.190536	0.053092
C	-3.646822	0.665866	1.346046
C	-2.448896	0.277575	1.906862
C	-1.243268	0.390095	1.184540
C	-1.296130	0.946997	-0.105764
C	-2.504019	1.327663	-0.654954
H	-4.561988	0.561635	1.915343
H	-2.429646	-0.140105	2.905165
H	-0.382708	1.100362	-0.664364
H	-2.525218	1.746758	-1.654249
C	7.359712	-2.689848	-1.599969
C	6.270503	-3.521533	-1.406861
C	5.095389	-3.010728	-0.881606
C	4.980274	-1.664564	-0.536908
H	8.293018	-3.080229	-1.985713
H	6.344303	-4.574571	-1.648808
H	4.251131	-3.665564	-0.704048
C	6.070950	-0.816429	-0.777028
C	7.253027	-1.335548	-1.308857
S	8.627387	-0.278162	-1.703358
S	5.921745	0.939962	-0.509975
C	8.667999	0.763453	-0.266470
C	7.488036	1.293095	0.250870
C	7.536725	2.141758	1.351903
C	8.757358	2.490265	1.908187
C	9.932482	1.961266	1.392394
C	9.887190	1.084607	0.319949
H	6.613879	2.532783	1.762288
H	8.788668	3.165117	2.754620
H	10.886806	2.220075	1.834153
H	10.797593	0.649034	-0.072738
C	-7.360408	2.688727	-1.601027
C	-6.271433	3.520800	-1.408274
C	-5.096168	3.010545	-0.882821
C	-4.980674	1.664555	-0.537570
H	-8.293829	3.078685	-1.986921

H	-6.345534	4.573717	-1.650655
H	-4.252094	3.665692	-0.705544
C	-6.071110	0.816011	-0.777327
C	-7.253339	1.334578	-1.309356
S	-8.627406	0.276641	-1.703399
S	-5.921407	-0.940226	-0.509549
C	-8.667705	-0.764386	-0.266075
C	-7.487586	-1.293481	0.251471
C	-7.536022	-2.141697	1.352859
C	-8.756549	-2.490312	1.909306
C	-9.931828	-1.961858	1.393309
C	-9.886798	-1.085635	0.320496
H	-6.613060	-2.532293	1.763394
H	-8.787658	-3.164818	2.756022
H	-10.886074	-2.220749	1.835190
H	-10.797328	-0.650482	-0.072359

S5.2 Py-2TA

S5.2.1 S_0

S	4.221525	3.384652	13.821604
S	6.810458	1.901203	14.979565
N	3.769858	7.360741	10.822033
C	4.758246	6.674857	11.382909
H	5.550234	7.257682	11.843795
C	4.813963	5.283238	11.417912
C	3.769858	4.586984	10.821997
H	3.769858	3.502697	10.821986
C	5.971390	4.594012	12.043486
C	5.829968	3.713455	13.125666
C	7.244733	4.823370	11.526746
H	7.349732	5.489083	10.678874
C	8.361059	4.210125	12.070513
H	9.343061	4.405076	11.657877
C	8.220389	3.350552	13.145763
H	9.085440	2.875149	13.590434
C	6.957266	3.087380	13.661533
C	5.711175	2.770254	16.069386
C	5.951461	2.774147	17.438968
H	6.837662	2.285905	17.824951
C	5.057901	3.393013	18.298476
H	5.241999	3.378637	19.365606
C	3.942808	4.045552	17.790601
H	3.251577	4.544166	18.458714
C	3.720325	4.077756	16.423235
H	2.866804	4.606203	16.017195
C	4.591916	3.422922	15.559454
S	3.318187	3.384651	7.822411
S	0.729241	1.901249	6.664423

C	2.781471	6.674872	10.261140
H	1.989483	7.257708	9.800268
C	2.725752	5.283253	10.226100
C	1.568323	4.594047	9.600509
C	1.709742	3.713484	8.518333
C	0.294977	4.823431	10.117230
H	0.189978	5.489147	10.965101
C	-0.821354	4.210207	9.573450
H	-1.803357	4.405178	9.986072
C	-0.680686	3.350629	8.498203
H	-1.545740	2.875241	8.053521
C	0.582439	3.087431	7.982452
C	1.828549	2.770281	5.574613
C	1.588278	2.774179	4.205029
H	0.702072	2.285952	3.819036
C	2.481857	3.393030	3.345530
H	2.297771	3.378658	2.278398
C	3.596956	4.045551	3.853417
H	4.288202	4.544153	3.185311
C	3.819426	4.077749	5.220785
H	4.672951	4.606182	5.626835
C	2.947815	3.422930	6.084557

S5.2.2 S_1

S	4.180486	3.365054	13.349665
S	6.878247	1.788144	14.042019
N	3.458332	7.928097	11.360500
C	4.491954	7.178764	11.720989
H	5.288478	7.681419	12.261734
C	4.591339	5.813345	11.453648
C	3.539571	5.218755	10.770537
H	3.566535	4.161711	10.532126
C	5.800975	5.056614	11.865083
C	5.742867	3.936390	12.707418
C	7.042347	5.470409	11.385545
H	7.084154	6.324659	10.720959
C	8.206104	4.806608	11.735708
H	9.161394	5.148064	11.356865
C	8.147018	3.708291	12.575395
H	9.049502	3.187814	12.869787
C	6.918118	3.261915	13.045665
C	5.785949	2.310487	15.340174
C	6.080667	1.999202	16.662923
H	7.002795	1.480902	16.894812
C	5.195143	2.345631	17.670921
H	5.422241	2.086151	18.697554
C	4.032211	3.040074	17.366660
H	3.346377	3.326511	18.154299
C	3.753892	3.386801	16.054199

H	2.861749	3.950487	15.811413
C	4.618997	3.005491	15.033957
S	2.849906	4.906388	7.539719
S	0.437480	2.561078	6.908091
C	2.461975	7.337946	10.709880
H	1.634095	7.973864	10.411645
C	2.438605	5.980825	10.390935
C	1.275417	5.379711	9.695865
C	1.451737	4.591137	8.516950
C	0.011588	5.579566	10.189014
H	-0.110768	6.141243	11.106853
C	-1.132001	5.028350	9.548088
H	-2.120179	5.242158	9.933286
C	-0.976721	4.189651	8.479195
H	-1.838629	3.705217	8.031621
C	0.303266	3.913265	7.967975
C	1.954997	2.676428	6.106018
C	2.189909	1.717180	5.093627
H	1.403841	1.007320	4.861051
C	3.370198	1.684579	4.410502
H	3.520312	0.952972	3.627079
C	4.397185	2.609795	4.723988
H	5.343998	2.566292	4.201399
C	4.195317	3.551934	5.689403
H	4.975801	4.264648	5.931085
C	2.978227	3.618661	6.408178

S5.2.3 T_1

S	4.175064	3.229412	12.596469
S	7.027764	1.766749	12.568954
N	2.888126	8.020690	12.129615
C	4.010565	7.297796	12.214130
H	4.771790	7.662599	12.895472
C	4.227083	6.128392	11.475989
C	3.229972	5.696170	10.627997
H	3.394136	4.828339	10.006817
C	5.528752	5.417135	11.571705
C	5.629290	4.100734	12.042285
C	6.685683	6.073074	11.158166
H	6.603339	7.085346	10.781661
C	7.921452	5.449809	11.211915
H	8.810708	5.977820	10.890917
C	8.018879	4.150591	11.678800
H	8.979429	3.654560	11.736998
C	6.874496	3.469623	12.075332
C	6.034402	1.771116	14.040599
C	6.475755	1.103451	15.177555
H	7.447395	0.625349	15.170428
C	5.672162	1.048446	16.305158

H	6.014097	0.513338	17.182414
C	4.443369	1.694309	16.315536
H	3.820435	1.666750	17.200935
C	4.017193	2.393918	15.197872
H	3.071612	2.921609	15.206382
C	4.801688	2.418280	14.049454
S	1.265384	3.224255	9.691496
S	0.611187	3.417271	6.531591
C	1.938603	7.609308	11.321096
H	1.047803	8.225371	11.289999
C	2.010253	6.422627	10.523771
C	0.946223	6.036022	9.696234
C	0.958769	4.771471	8.903859
C	-0.116598	6.983834	9.303259
H	-0.529420	7.684544	10.016357
C	-0.498869	7.004524	8.013869
H	-1.206793	7.745564	7.661201
C	-0.018407	6.014799	7.087148
H	-0.266691	6.084878	6.036308
C	0.585312	4.844975	7.586993
C	2.051115	2.559443	7.119358
C	2.891331	1.938589	6.201738
H	2.690353	2.042070	5.142603
C	3.972474	1.190685	6.641746
H	4.612646	0.698474	5.920082
C	4.242859	1.090118	7.998801
H	5.094875	0.519701	8.346928
C	3.431269	1.736682	8.918540
H	3.653124	1.684758	9.977764
C	2.321564	2.452634	8.481705

S5.3 PyCN-2TA

S5.2.1 S_0

S	2.997371	12.573232	10.587591
S	0.949091	10.728460	8.972613
S	3.456342	19.234355	9.360042
S	6.186888	20.540735	8.337520
N	2.374960	16.570112	13.269164
N	3.687556	15.539124	8.176852
C	1.648572	15.743720	12.528154
H	0.786387	15.291378	13.007768
C	1.934445	15.427855	11.203193
C	3.055367	16.035416	10.630188
C	3.832449	16.913005	11.391649
C	3.437252	17.132438	12.707989
H	4.015506	17.807852	13.330445
C	3.406548	15.759643	9.267542
C	1.417030	13.218108	10.085139

C	1.034346	14.515042	10.447673
C	-0.246070	14.958890	10.128690
H	-0.542799	15.958564	10.421501
C	-1.123647	14.150185	9.424784
H	-2.110334	14.514963	9.168366
C	-0.731447	12.882111	9.033260
H	-1.400119	12.249360	8.463374
C	0.527216	12.406144	9.380387
C	2.613975	10.945586	8.391869
C	3.037512	10.280932	7.246872
H	2.334247	9.670665	6.694064
C	4.351347	10.400738	6.822602
H	4.679693	9.869551	5.937809
C	5.234382	11.218350	7.513839
H	6.254753	11.332427	7.169897
C	4.806311	11.912966	8.633786
H	5.479341	12.579328	9.157753
C	3.502584	11.758915	9.090149
C	5.019187	18.598679	9.924921
C	5.063253	17.565237	10.868234
C	6.296062	17.154917	11.368270
H	6.325292	16.363319	12.106957
C	7.473238	17.726978	10.913726
H	8.426001	17.385856	11.298744
C	7.431486	18.722301	9.953135
H	8.345602	19.161287	9.573722
C	6.207840	19.174634	9.474226
C	4.969687	19.974630	7.173807
C	5.192113	20.132927	5.810855
H	6.126609	20.560526	5.469567
C	4.220676	19.746568	4.901148
H	4.393328	19.885089	3.841041
C	3.043675	19.161116	5.346072
H	2.293858	18.836355	4.635600
C	2.834010	18.966275	6.701836
H	1.934067	18.479048	7.054355
C	3.784960	19.395110	7.620261

S5.3.2 S_1

S	3.009752	13.045169	10.237416
S	1.033175	11.301547	8.068975
S	3.576604	19.305961	9.339027
S	6.405130	20.331964	8.248005
N	2.230376	16.834841	13.179644
N	3.232289	15.641051	8.027753
C	1.463178	16.023352	12.495745
H	0.574036	15.661944	13.009118
C	1.725862	15.539122	11.201063
C	2.899526	16.055380	10.535949

C	3.746145	16.913610	11.296755
C	3.378593	17.250035	12.570869
H	4.014941	17.902255	13.158469
C	3.106266	15.858336	9.159790
C	1.367702	13.430621	9.867865
C	0.849290	14.551623	10.610778
C	-0.541666	14.646455	10.691324
H	-0.971873	15.466874	11.250686
C	-1.372910	13.786921	9.994302
H	-2.445746	13.924454	10.050402
C	-0.860189	12.796757	9.162199
H	-1.516086	12.188817	8.552979
C	0.515850	12.609399	9.109739
C	2.774348	11.333078	8.045948
C	3.384555	10.541706	7.065264
H	2.764811	10.030972	6.338561
C	4.753821	10.417649	7.017048
H	5.207493	9.805941	6.247873
C	5.557874	11.082649	7.946799
H	6.634964	10.993058	7.899798
C	4.977160	11.868968	8.910804
H	5.589824	12.412088	9.619542
C	3.581189	12.006733	8.972102
C	5.077703	18.484532	9.825348
C	5.029270	17.426426	10.739903
C	6.225244	16.861158	11.173907
H	6.184936	16.056608	11.898011
C	7.445727	17.306702	10.690714
H	8.366073	16.849492	11.033168
C	7.487892	18.330029	9.759832
H	8.432710	18.674384	9.358283
C	6.307958	18.934094	9.343267
C	5.089362	19.950783	7.117474
C	5.276642	20.135455	5.752311
H	6.239904	20.470124	5.387579
C	4.234694	19.894548	4.871090
H	4.381226	20.054576	3.809873
C	3.018006	19.424292	5.345678
H	2.210507	19.209548	4.656664
C	2.839980	19.201855	6.701545
H	1.909008	18.796710	7.076121
C	3.864874	19.490357	7.595644

S5.3.3 T_1

S	2.711421	12.392735	10.423290
S	0.447108	11.181814	8.509166
S	3.240444	18.705349	9.399421
S	5.426260	20.777361	8.337267
N	3.156575	15.728301	13.409621

N	4.443815	15.357425	8.314945
C	2.187554	15.307235	12.487893
H	1.316490	14.836682	12.925929
C	2.330846	15.369925	11.128846
C	3.496222	15.979368	10.610658
C	4.331426	16.763215	11.568581
C	4.169068	16.374467	12.959913
H	4.881689	16.733636	13.697426
C	4.008379	15.630112	9.350346
C	1.392086	13.424397	9.824105
C	1.312631	14.760548	10.227105
C	0.245964	15.540917	9.792028
H	0.184567	16.572028	10.118076
C	-0.720898	15.013466	8.950380
H	-1.543557	15.631883	8.613415
C	-0.627885	13.697398	8.529738
H	-1.365749	13.280650	7.855739
C	0.414966	12.896290	8.978437
C	2.166621	10.995150	8.101707
C	2.532551	10.288094	6.962105
H	1.763093	9.892931	6.310562
C	3.872664	10.091381	6.669118
H	4.151982	9.527180	5.787869
C	4.851170	10.637841	7.487788
H	5.898840	10.506768	7.247386
C	4.490757	11.375738	8.603797
H	5.246906	11.835040	9.227359
C	3.148491	11.535095	8.928646
C	4.862469	18.709368	10.088776
C	5.145299	17.860578	11.206957
C	6.331630	18.147255	11.945853
H	6.565213	17.555144	12.819311
C	7.231774	19.081303	11.508693
H	8.152706	19.237552	12.056496
C	6.992673	19.814870	10.342003
H	7.718373	20.534459	9.985620
C	5.793907	19.651444	9.660474
C	4.567032	19.714788	7.203758
C	4.827530	19.805041	5.842239
H	5.591375	20.486299	5.488854
C	4.109312	19.026228	4.947631
H	4.308256	19.109468	3.886397
C	3.159195	18.127485	5.409953
H	2.615988	17.501267	4.713741
C	2.919130	18.007733	6.769790
H	2.207506	17.281314	7.140061
C	3.606137	18.818987	7.663730

S5.4 Pm-2TA

S5.4.1 S_0

S	1.816932	3.386181	16.245265
S	1.021865	3.691161	13.150847
S	2.815948	-1.607063	4.435029
S	3.692895	0.773408	6.400854
N	2.548811	2.917682	10.757728
N	2.179858	2.020508	8.595396
C	-0.223364	3.544981	14.414789
C	-1.562943	3.606822	14.043391
H	-1.819726	3.673761	12.993368
C	-2.556023	3.593314	15.009828
H	-3.595362	3.658907	14.712525
C	-2.219149	3.478613	16.351708
H	-2.993604	3.454136	17.108357
C	-0.888884	3.376256	16.726026
H	-0.617550	3.261237	17.768135
C	0.110522	3.429046	15.760926
C	2.461996	2.266584	15.022833
C	3.324685	1.262294	15.447717
H	3.527405	1.152336	16.505526
C	3.921750	0.422951	14.524891
H	4.609672	-0.345594	14.853941
C	3.635732	0.572467	13.178829
H	4.117950	-0.068389	12.451018
C	2.756972	1.556932	12.734533
C	2.159804	2.419552	13.667639
C	2.521180	1.689030	11.276713
C	2.306949	0.576760	10.471512
H	2.276711	-0.419457	10.890016
C	2.380951	3.016038	9.446792
H	2.411263	4.016130	9.026678
C	2.132891	0.790322	9.109356
C	1.858794	-0.330108	8.177785
C	2.479464	-0.424564	6.921902
C	0.920476	-1.289118	8.550121
H	0.421574	-1.192178	9.506485
C	0.597654	-2.339333	7.708173
H	-0.136394	-3.075187	8.011262
C	1.217325	-2.442780	6.476143
H	0.986114	-3.264014	5.809616
C	2.140201	-1.482936	6.075718
C	4.532404	-1.320214	4.781087
C	5.506451	-2.098135	4.165387
H	5.206871	-2.908383	3.512247
C	6.848076	-1.828630	4.383562
H	7.603549	-2.426263	3.888595
C	7.219599	-0.807947	5.248122
H	8.267172	-0.603920	5.432189

C	6.249849	-0.056856	5.892982
H	6.532584	0.726230	6.585660
C	4.901099	-0.295572	5.647800

S5.4.2 S_1

N	1.011452	-1.327787	-0.939179
C	-0.180204	-1.077356	-1.461963
N	-1.191050	-0.439654	-0.904511
C	-1.020733	0.035298	0.361425
C	0.211049	-0.213244	1.014572
C	1.189167	-0.891114	0.331143
H	-0.341864	-1.440591	-2.474059
H	0.386681	0.108576	2.031076
C	-4.113500	2.571640	1.895444
C	-2.098543	0.782976	0.944481
C	-1.867885	1.693624	1.983115
C	-2.836713	2.563626	2.450357
H	-4.868895	3.267744	2.233283
H	-0.869479	1.765185	2.392235
H	-2.591688	3.267095	3.236156
C	4.888275	-1.928851	2.210890
C	2.491345	-1.212265	0.964277
C	2.502440	-1.758222	2.245446
C	3.686963	-2.110472	2.869826
H	5.825147	-2.194315	2.684104
H	1.557308	-1.931116	2.744861
H	3.672900	-2.534369	3.866376
C	-4.420495	1.623755	0.919050
C	-3.467517	0.705566	0.455244
S	-3.830077	-0.663972	-0.565370
S	-6.069541	1.773163	0.349935
C	-5.557686	-0.750403	-0.702024
C	-6.490138	0.238751	-0.343428
C	-7.854817	0.024714	-0.588679
C	-8.284856	-1.136897	-1.180744
C	-7.359443	-2.125030	-1.547519
C	-6.023572	-1.932417	-1.312940
H	-8.568568	0.785822	-0.297461
H	-9.341957	-1.288894	-1.357180
H	-7.697020	-3.042650	-2.011452
H	-5.304424	-2.693889	-1.590184
C	4.900330	-1.410793	0.920363
C	3.712680	-1.032179	0.291621
S	3.760314	-0.312016	-1.340730
S	6.465446	-1.314706	0.078103
C	5.179938	0.751364	-1.187861
C	6.362018	0.325053	-0.589796
C	7.467249	1.167849	-0.543860
C	7.407659	2.422152	-1.129271

C	6.230391	2.852719	-1.726348
C	5.116416	2.028755	-1.737954
H	8.372469	0.831625	-0.053213
H	8.276289	3.068785	-1.105726
H	6.174251	3.838520	-2.171742
H	4.187469	2.367299	-2.180231

S5.4.3 T_1

N	1.160345	-1.424687	-0.944117
C	-0.026290	-1.213301	-1.507661
N	-1.074229	-0.627259	-0.968063
C	-0.951777	-0.164564	0.301980
C	0.274924	-0.340875	0.979952
C	1.298977	-0.985266	0.316713
H	-0.141074	-1.566627	-2.528340
H	0.432419	0.032993	1.980506
C	-4.073218	2.403179	1.598479
C	-2.082865	0.484945	0.868817
C	-2.051697	1.124701	2.141863
C	-2.981495	2.052026	2.484260
H	-4.748961	3.204621	1.861071
H	-1.235915	0.923257	2.823514
H	-2.910992	2.554603	3.441390
C	5.003674	-1.814406	2.278300
C	2.604037	-1.239822	0.975917
C	2.612095	-1.757807	2.268109
C	3.800060	-2.043961	2.919312
H	5.942790	-2.022382	2.775275
H	1.666560	-1.958901	2.756463
H	3.787676	-2.447991	3.923863
C	-4.270351	1.667487	0.475360
C	-3.379400	0.610753	0.130332
S	-3.887099	-0.754542	-0.788920
S	-5.628943	2.071704	-0.600280
C	-5.659530	-0.695404	-0.776043
C	-6.402610	0.484406	-0.732294
C	-7.789831	0.423865	-0.835805
C	-8.428669	-0.792310	-1.002454
C	-7.689683	-1.968557	-1.042791
C	-6.313265	-1.919085	-0.924315
H	-8.363920	1.340656	-0.781073
H	-9.507819	-0.823524	-1.087303
H	-8.186330	-2.923447	-1.159482
H	-5.729786	-2.831890	-0.948418
C	5.017621	-1.321485	0.978157
C	3.824518	-1.018292	0.318563
S	3.867866	-0.348660	-1.333756
S	6.591096	-1.156246	0.164449
C	5.216586	0.803185	-1.180462

C	6.407537	0.458394	-0.547750
C	7.460712	1.364859	-0.499189
C	7.340600	2.600177	-1.115402
C	6.154047	2.948356	-1.746487
C	5.089655	2.061135	-1.761996
H	8.373145	1.093811	0.017475
H	8.169639	3.296630	-1.089459
H	6.050906	3.918834	-2.216153
H	4.153244	2.335166	-2.232156

S5.5 PmCN-2TA

S5.5.1 S_0

N	-1.019458	0.601467	-1.832767
C	0.000065	0.000524	-2.427423
N	1.019470	-0.600509	-1.832683
C	1.024587	-0.624522	-0.503436
C	-0.000123	0.000372	0.225188
C	-1.024732	0.625324	-0.503529
H	0.000155	0.000605	-3.512367
C	-0.000232	0.000389	1.654734
C	4.189963	-2.822303	1.323558
C	2.143060	-1.352612	0.144282
C	1.856237	-2.362841	1.056317
C	2.872799	-3.098618	1.641014
H	4.996043	-3.374809	1.789614
H	0.825512	-2.579165	1.305688
H	2.636934	-3.880035	2.351838
C	-4.190593	2.822385	1.323495
C	-2.143364	1.353210	0.144176
C	-1.856746	2.363557	1.056139
C	-2.873481	3.099091	1.640842
H	-4.996792	3.374683	1.789593
H	-0.826060	2.580124	1.305471
H	-2.637797	3.880613	2.351611
C	4.493411	-1.832700	0.394843
C	3.476274	-1.087471	-0.202731
S	3.883521	0.180899	-1.386346
S	6.196586	-1.591047	-0.055387
C	5.269965	0.945048	-0.572620
C	6.286223	0.181086	-0.005886
C	7.385221	0.805114	0.573446
C	7.488112	2.186857	0.552301
C	6.475348	2.950795	-0.011311
C	5.360358	2.332960	-0.554949
H	8.158089	0.202767	1.034478
H	8.353836	2.667506	0.990804
H	6.545860	4.031436	-0.015613
H	4.556119	2.923469	-0.976042

C	-4.493824	1.832645	0.394860
C	-3.476507	1.087701	-0.202758
S	-3.883385	-0.180683	-1.386486
S	-6.196970	1.590396	-0.055176
C	-5.269537	-0.945375	-0.572768
C	-6.285980	-0.181777	-0.005875
C	-7.384747	-0.806208	0.573468
C	-7.487225	-2.187979	0.552173
C	-6.474275	-2.951549	-0.011603
C	-5.359510	-2.333315	-0.555250
H	-8.157761	-0.204149	1.034629
H	-8.352771	-2.668938	0.990687
H	-6.544461	-4.032210	-0.016028
H	-4.555125	-2.923533	-0.976473
N	-0.000340	0.000455	2.802912

S5.5.2 S_1

N	-1.027547	0.854739	-1.095869
C	0.142402	0.611572	-1.680163
N	1.219521	0.087935	-1.149069
C	1.160736	-0.286197	0.168169
C	-0.052425	-0.041150	0.913234
C	-1.139451	0.546784	0.219689
H	0.212017	0.874839	-2.731377
C	-0.153034	-0.371281	2.276624
C	4.497809	-2.714334	1.389252
C	2.304463	-1.014104	0.642710
C	2.181740	-2.054679	1.579642
C	3.230967	-2.872294	1.941979
H	5.317545	-3.354674	1.682217
H	1.212866	-2.258627	2.005435
H	3.060436	-3.668055	2.656543
C	-4.683992	2.095249	2.061547
C	-2.386037	0.980757	0.842233
C	-2.340385	1.633155	2.082165
C	-3.450852	2.178034	2.679692
H	-5.562862	2.513199	2.533079
H	-1.389794	1.751047	2.577878
H	-3.356478	2.682175	3.633706
C	4.702196	-1.673276	0.493882
C	3.652866	-0.797730	0.130172
S	3.890854	0.599788	-0.875776
S	6.322924	-1.632211	-0.166733
C	5.599260	0.958741	-0.804051
C	6.622978	0.048377	-0.507333
C	7.955790	0.458754	-0.580071
C	8.266742	1.753642	-0.937996
C	7.251702	2.663318	-1.239646
C	5.934862	2.266635	-1.180783

H	8.743626	-0.243756	-0.337978
H	9.303440	2.063555	-0.974816
H	7.495958	3.681878	-1.511763
H	5.140743	2.967178	-1.408277
C	-4.795915	1.445684	0.838672
C	-3.682624	0.860754	0.213375
S	-3.867306	-0.084583	-1.268298
S	-6.411498	1.501040	0.129519
C	-5.505425	-0.737305	-1.168266
C	-6.589627	-0.085089	-0.590923
C	-7.862170	-0.653052	-0.648285
C	-8.060146	-1.857022	-1.296928
C	-6.982014	-2.513117	-1.881965
C	-5.717418	-1.959754	-1.810784
H	-8.694916	-0.142699	-0.179294
H	-9.051878	-2.290583	-1.337803
H	-7.129442	-3.462194	-2.382140
H	-4.870211	-2.469060	-2.253917
N	-0.193206	-0.659134	3.395520

S5.5.3 T_1

N	-0.964853	1.323471	-1.314209
C	0.253538	0.999928	-1.756627
N	1.214901	0.425439	-1.080946
C	0.994319	0.079928	0.217547
C	-0.257392	0.471307	0.804654
C	-1.200933	1.084270	-0.024839
H	0.462975	1.239805	-2.795138
C	-0.552953	0.295136	2.184316
C	4.047617	-2.532250	1.689435
C	2.045579	-0.649328	0.845749
C	1.867126	-1.461519	1.983487
C	2.814292	-2.369119	2.381679
H	4.748545	-3.298927	1.988242
H	0.935347	-1.430631	2.525768
H	2.607743	-3.000348	3.237183
C	-4.989549	2.474394	1.376895
C	-2.528842	1.531623	0.475828
C	-2.598485	2.454535	1.512775
C	-3.820848	2.927061	1.961119
H	-5.952908	2.818431	1.731697
H	-1.684049	2.806322	1.972457
H	-3.861084	3.640639	2.774232
C	4.342675	-1.681266	0.667601
C	3.415920	-0.669618	0.263208
S	3.890733	0.741035	-0.595350
S	5.841708	-1.980925	-0.212434
C	5.647451	0.763326	-0.607199
C	6.465335	-0.362863	-0.503130

C	7.844646	-0.225758	-0.670911
C	8.398470	1.008598	-0.942273
C	7.584802	2.136005	-1.038132
C	6.222716	2.012552	-0.872264
H	8.477517	-1.099621	-0.574904
H	9.470222	1.098977	-1.066807
H	8.017899	3.107497	-1.238575
H	5.581531	2.883021	-0.946158
C	-4.936324	1.573583	0.318994
C	-3.709448	1.093486	-0.139154
S	-3.653515	-0.060414	-1.496324
S	-6.459796	1.103493	-0.469632
C	-4.988879	-1.147173	-1.046221
C	-6.214725	-0.648713	-0.612893
C	-7.257324	-1.521288	-0.321640
C	-7.090400	-2.885348	-0.499572
C	-5.869020	-3.385086	-0.930360
C	-4.815989	-2.520716	-1.184020
H	-8.198100	-1.124564	0.039455
H	-7.910861	-3.559312	-0.286034
H	-5.729982	-4.451904	-1.055115
H	-3.853017	-2.905024	-1.496837
N	-0.778348	0.158299	3.304595

S6 Further results from experimental photophysical characterization

This section contains further results from the experimental photophysical characterization of the RTP emitter molecules, i.e. their absorption spectra in DCM in Fig. S27, their emission properties in PS using λ_{exc} = 300 nm in Figs S28 and S29, and the emission properties of the reference material **BP-2TA** in Fig. S30. Low temperature delayed spectra are provided in Fig. S31. This is followed by an extended analysis of the most promising emitter **Py-2TA** in comparison with **BP-2TA** in solvents of different polarity (Fig. S32), at different concentrations (Fig. S33) and in neat film (Fig. S34).

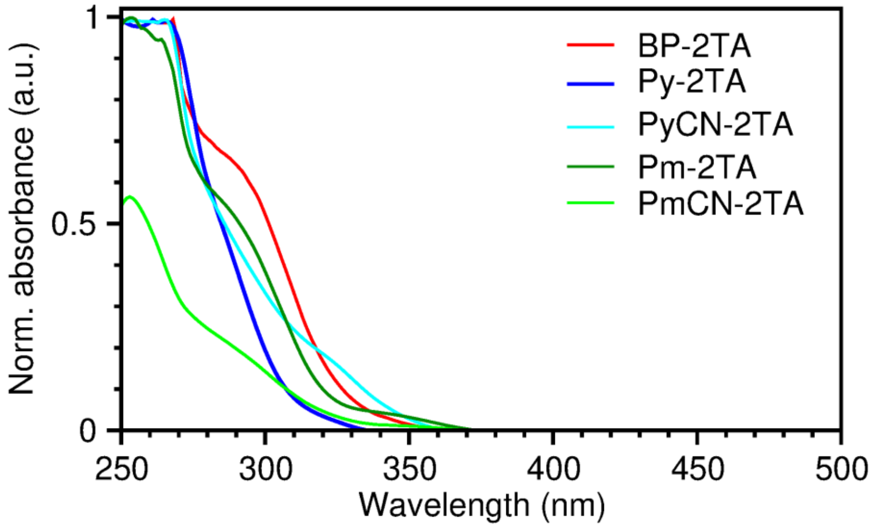

Figure S27. Absorption spectra of **BP-2TA**, **Py-2TA**, **PyCN-2TA**, **Pm-2TA**, and **PmCN-2TA** in DCM.

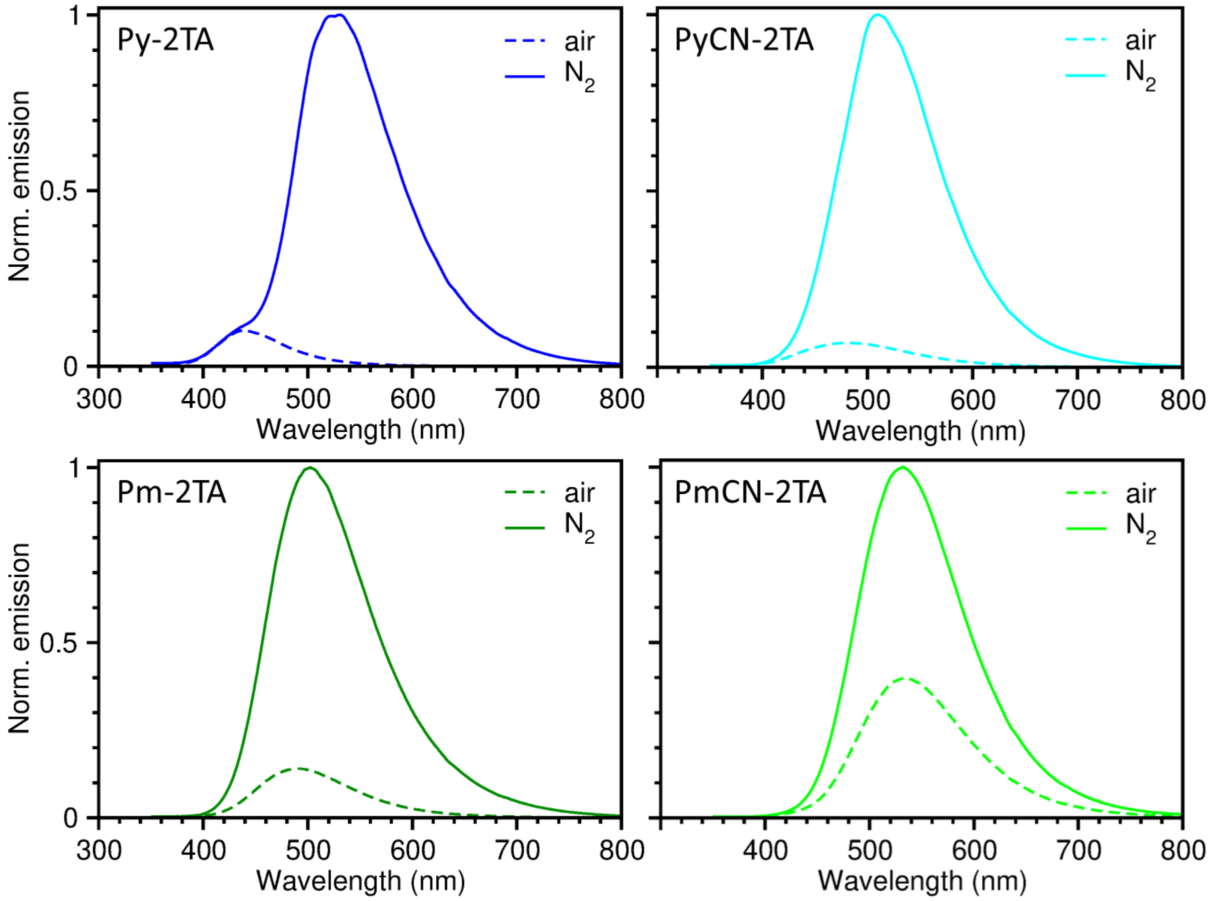

Figure S28. Emission spectra (λ_{exc} = 300 nm) of **Py-2TA**, **PyCN-2TA**, **Pm-2TA**, and **PmCN-2TA** in PS at room temperature under aerated (dashed lines) and nitrogen atmosphere.

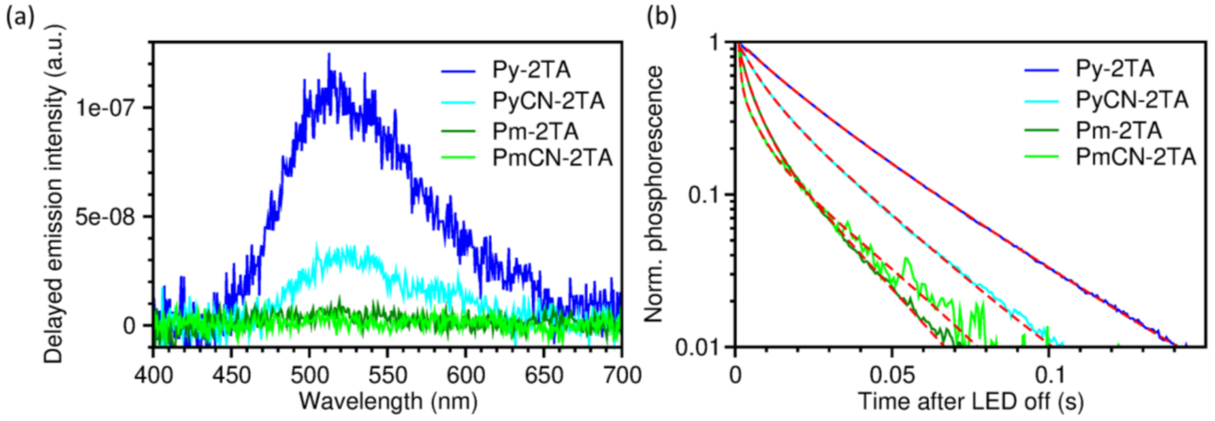

Figure S29. Delayed spectra (a) and corresponding phosphorescence decay (b) of **Py-2TA**, **PyCN-2TA**, **Pm-2TA**, and **PmCN-2TA** in PS at room temperature under nitrogen atmosphere collected at a delay time of 10 ms showing only the phosphorescence (λ_{exc} = 300 nm). Biexponential fit functions (**Py-2TA**, **PyCN-2TA**) and triexponential fit functions (**Pm-2TA**, **PmCN-2TA**) used to extract the phosphorescence lifetimes are presented as red dashed lines in (b). Difference regarding Fig. 6 in the main manuscript is the excitation wavelength. Only subtle differences in the emission properties are observed as shown in Tab. 2.

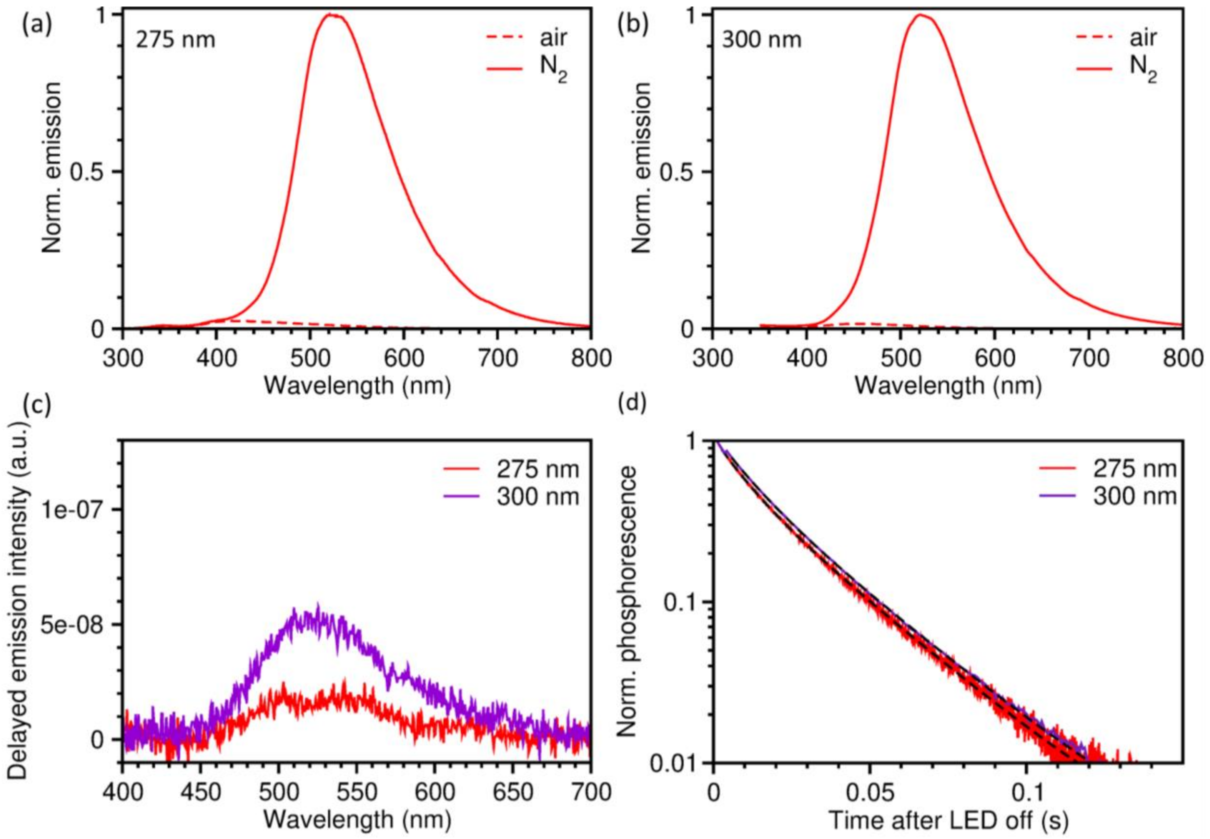

Figure S30. Photophysical properties of **BP-2TA** in PS at room temperature. Emission spectra with $\lambda_{\text{exc}} = 275$ nm (a) and $\lambda_{\text{exc}} = 300$ nm (b) under aerated (dashed lines) and nitrogen atmosphere. Delayed spectra (c) and corresponding phosphorescence decay (d) under nitrogen atmosphere collected at a delay time of 10 ms showing only the phosphorescence. Biexponential fit functions used to extract the phosphorescence lifetimes are presented as black dashed lines in (d).

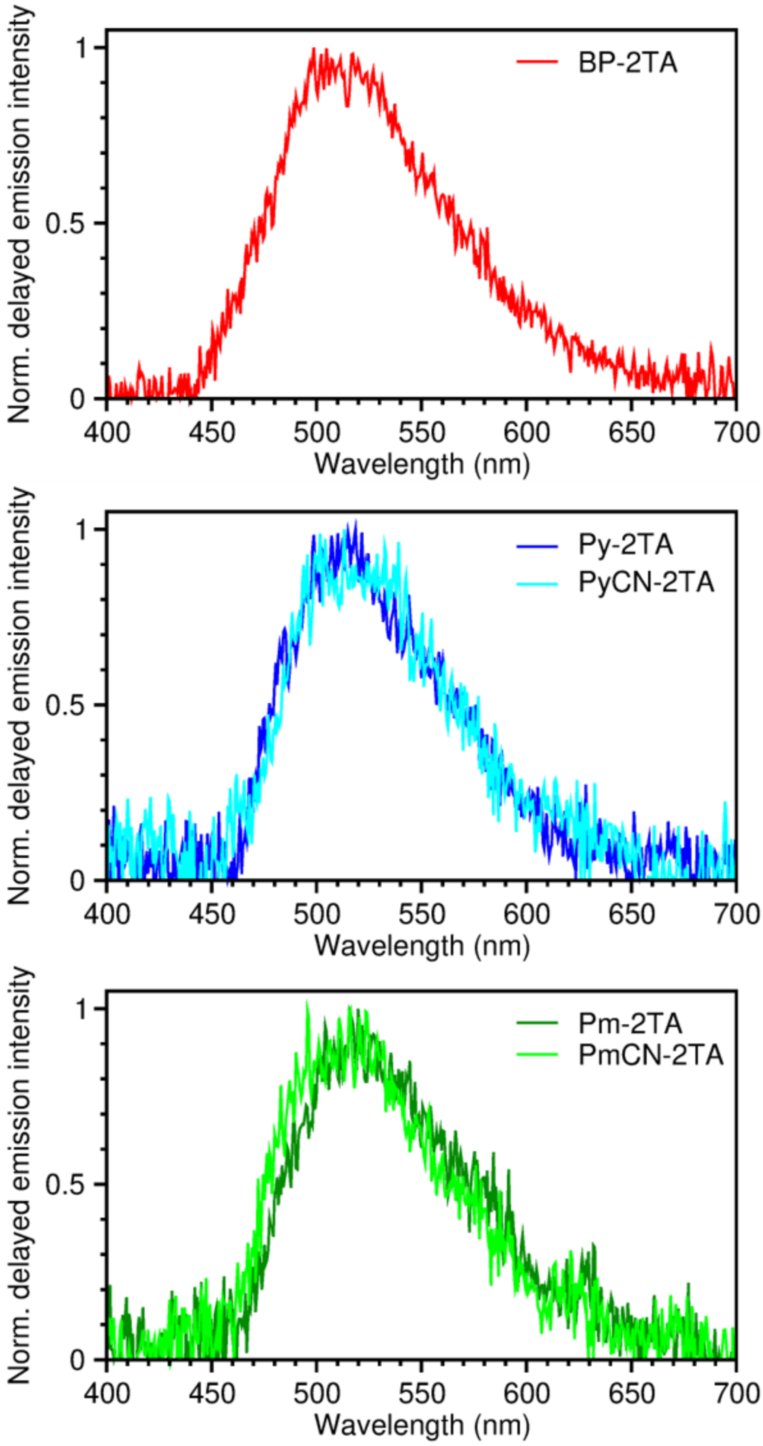

Figure S31. Delayed spectra of **BP-2TA**, **Py-2TA**, **PyCN-2TA**, **Pm-2TA**, and **PmCN-2TA** in PS (5 wt%) at 77 K collected at a delay time of 10 ms showing only the phosphorescence (λ_{exc} = 275 nm).

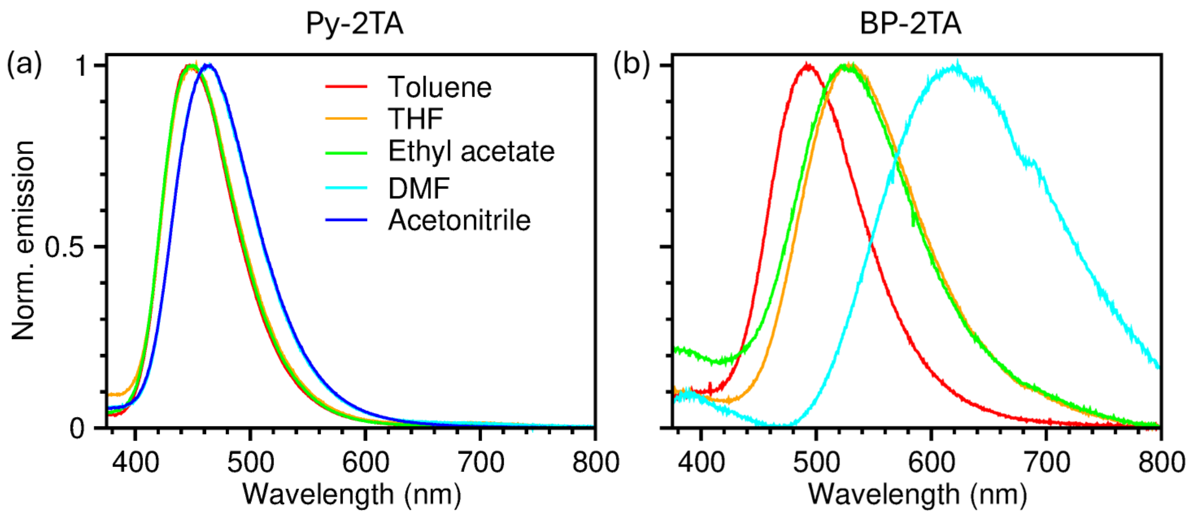

Figure S32. Emission spectra (λ_{exc} = 275 nm) of **Py-2TA** (a) and **BP-2TA** (b) in solvents of increasing polarity with toluene being least polar and acetonitrile being most polar. For **BP-2TA** the solubility in acetonitrile was not sufficient to measure reliable emission spectra. While **Py-2TA** shows only a minor shift by increasing the polarity of the solvent, **BP-2TA** shows a relatively strong red shift indicating its much more pronounced CT-state character of the $S_1 \rightarrow S_0$ transition.

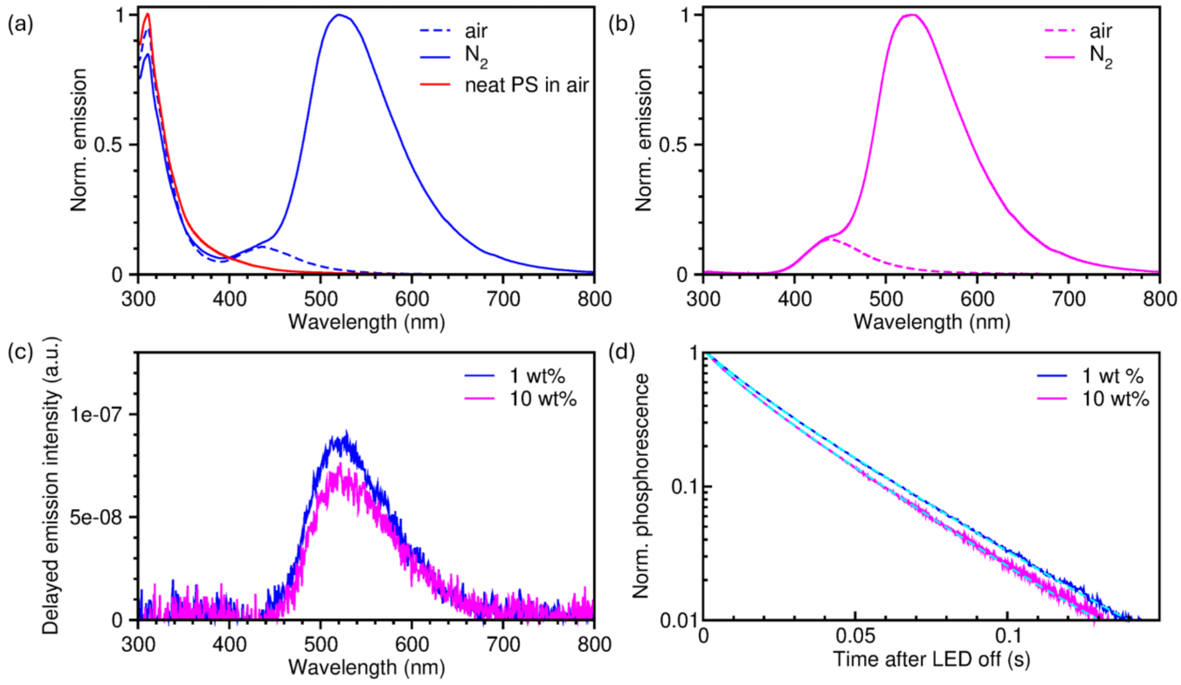

Figure S33. Emission spectra (λ_{exc} = 275 nm) of pure PS (a) and films of **Py-2TA** (1 wt% (a) and 10 wt% (b)) in PS at room temperature under aerated (dashed lines) and nitrogen atmosphere (solid lines). The pronounced emission peak around 310 nm can be assigned to the PS host. Delayed spectra (c) and corresponding phosphorescence decay (d) under nitrogen atmosphere collected at a delay time of 10 ms showing only the phosphorescence. Biexponential fit functions used to extract the phosphorescence lifetimes are presented as cyan dashed lines in (d). Key characteristics are summarized in Tab. S6.

Table S6. Emission properties of **Py-2TA** in PS at different emitter concentration at room temperature. When the emitter concentration is increased, no significant changes in the photophysical properties are observed, indicating sufficient intermixing and negligible emitter interactions even at elevated concentrations of 10 wt%. Due to relatively pronounced emission by PS in the 1 wt% samples, no reasonable Weber contrast K_W can be defined.

Compound	λ_{exc}[a], nm	λ_{max} in air[b], nm	λ_{max} in N_2[c], nm	K_W[d]	τ_P[e], ms	PLQY in air/N_2[f], %
**1%** of **Py-2TA**	275	435	520	-	29	5/23
**5%** of **Py-2TA**	275	440	530	15.1	28	2/25
**10%** of **Py-2TA**	275	435	520	11.7	27	3/22

[a] Excitation wavelength. [b] Wavelength of the emission maximum under aerated atmosphere. [c] Wavelength of the emission maximum under N_2 atmosphere. [d] Weber contrast. [e] Phosphorescence lifetime extracted from delayed spectroscopy (cf. Figs. S33d and Fig.6b) via biexponential fits. [f] PLQY under aerated and N_2 atmosphere.

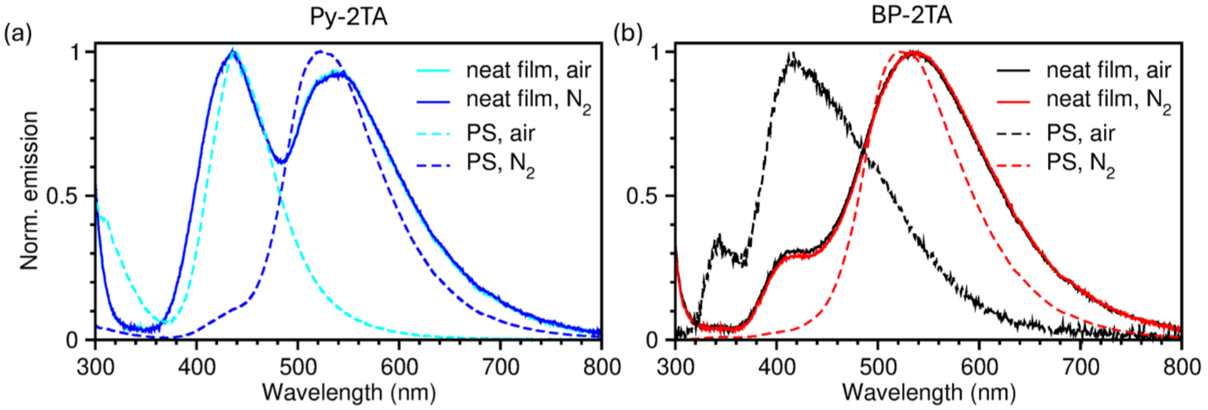

Figure S34. Emission spectra (λ_{exc} = 275 nm) of evaporated neat films of **Py-2TA** (a) and **BP-2TA** (b) at room temperature under aerated (dashed lines) and nitrogen atmosphere and comparison with diluted systems in PS (5 wt%). In neat films, RTP emission already occurs under aerated atmosphere due to a reduced phosphorescence lifetime and, thus, a reduced oxygen sensitivity. The RTP intensity is strongly reduced due to triplet-triplet annihilation and a potentially insufficiently rigid molecular environment.

S7 Characterization of programmable luminescent tags

This section contains further information on the experimental setup used to characterize the activation behavior or programmable luminescent tags (PLTs) as well as additional results.

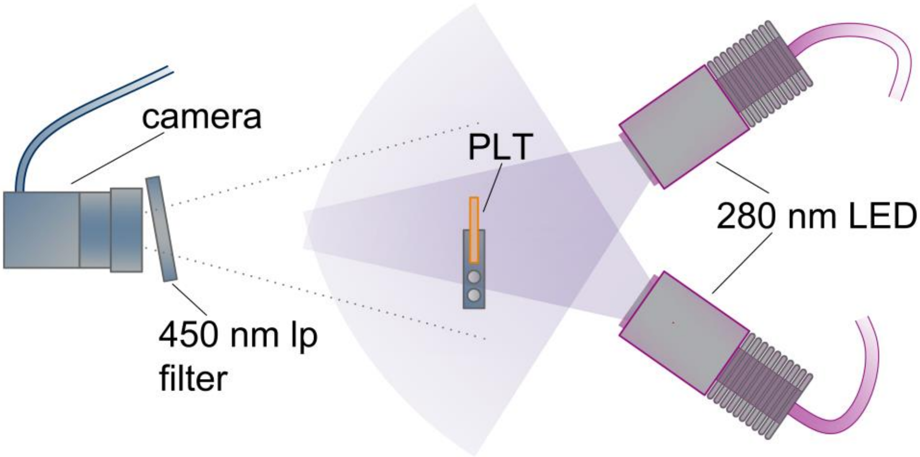

Figure S35. Schematic of the set-up for recording activation curves. The PLT is homogeneously illuminated by two identical LEDs whiles direct illumination of the camera is prohibited. In addition, a 450 nm long-pass (lp) filter protects the camera sensor from overexposure.

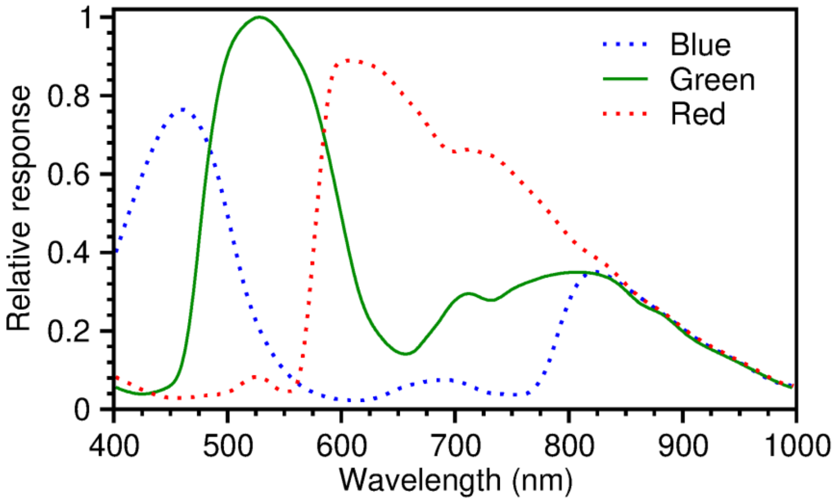

Figure S36. Spectral response curves of the CMOS camera acA1920-40uc from Basler. The green channel is used for characterizing the activation behavior of PLTs. Adapted from Ref. 1.

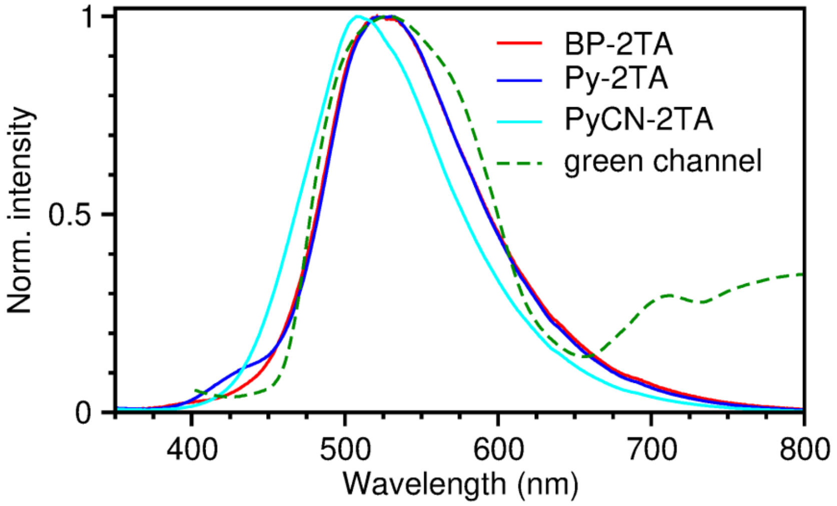

Figure S37. Emission spectra ($\lambda_{\mathrm{exc}} = 275$ nm) of **BP-2TA**, **Py-2TA**, and **PyCN-2TA** in PS at room temperature under nitrogen atmosphere versus spectral response curve of the green channel of the camera (dashed line).

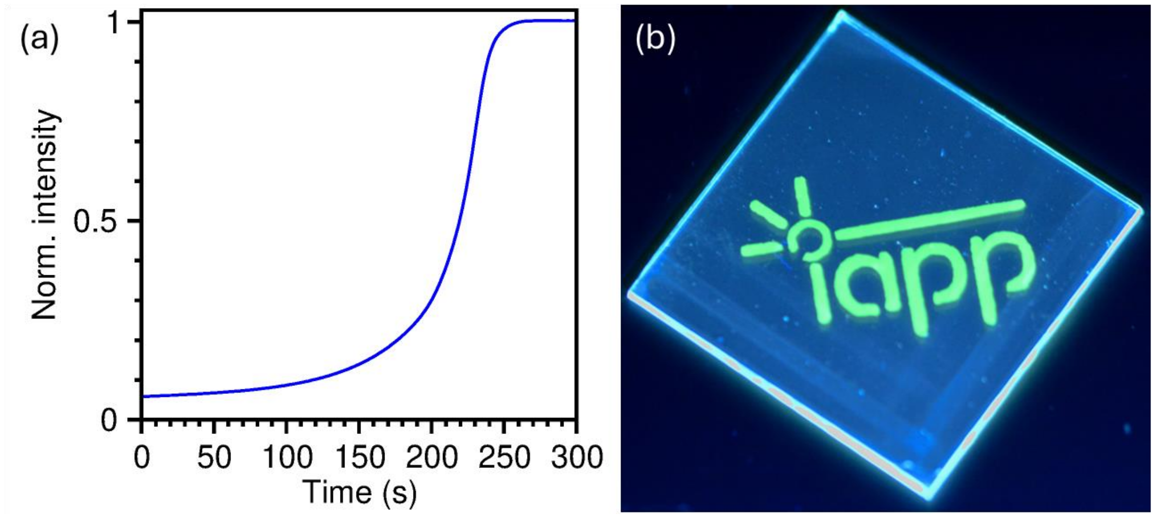

Figure S38. Performance of PLTs using **Py-2TA** as RTP emitter (5 wt%) and PMMA as host. (a) Photoluminescence intensity over illumination time for the first activation cycle. (b) Photo of a locally activated PLT.

References

1. Basler ace USB 3.0 camera (cA1920-40uc). www.baslerweb.com (2023).
