# Supplementary material for: Systematic variation of the acceptor electrophilicity in donor-acceptor-donor emitters exhibiting efficient room temperature phosphorescence suited for digital luminescence
Source: Commun Chem. 2025 Sep 10;8:274. doi: 10.1038/s42004-025-01620-0 (PMC12423317; doi:10.1038/s42004-025-01620-0)
Supplement: Supplementary file 3 — Description of Additional Supplementary Files [file 42004_2025_1620_MOESM3_ESM.pdf]

## Description of Additional Supplementary Files

File name- Supplementary Data 1

File description – CIF report for BP-2TA

File name- Supplementary Data 2

File description – CIF report for Py-2TA

File name- Supplementary Data 3

File description – CIF report for PyCN-2TA

File name- Supplementary Data 4
File description – CIF report for Pm-2TA.
