# Supplementary material for: Systematic variation of the acceptor electrophilicity in donor-acceptor-donor emitters exhibiting efficient room temperature phosphorescence suited for digital luminescence
Source: Commun Chem. 2025 Sep 10;8:274. doi: 10.1038/s42004-025-01620-0 (PMC12423317; doi:10.1038/s42004-025-01620-0)

checkCIF/PLATON report

Structure factors have been supplied for datablock(s) iapp_utd1_final

THIS REPORT IS FOR GUIDANCE ONLY. IF USED AS PART OF A REVIEW PROCEDURE FOR PUBLICATION, IT SHOULD NOT REPLACE THE EXPERTISE OF AN EXPERIENCED CRYSTALLOGRAPHIC REFEREE.

No syntax errors found.   CIF dictionary   Interpreting this report

Datablock: iapp_utd1_final

Bond precision: C-C = 0.0019 A   Wavelength=1.54184

Cell:	a=15.13566(11)	b=6.95148(4)	c=21.64403(15)
	alpha=90	beta=90.0748(6)	gamma=90
Temperature:	100 K		

	Calculated	Reported
Volume	2277.28(3)	2277.28(3)
Space group	I 2/a	I 1 2/a 1
Hall group	-I 2ya	-I 2ya
Moiety formula	C29 H17 N S4	C29 H17 N S4
Sum formula	C29 H17 N S4	C29 H17 N S4
Mr	507.68	507.67
Dx,g cm-3	1.481	1.481
Z	4	4
Mu (mm-1)	3.982	3.982
F000	1048.0	1048.0
F000'	1055.48	
h,k,lmax	19,8,27	19,8,27
Nref	2405	2388
Tmin,Tmax	0.592,0.887	0.573,0.897
Tmin'	0.397	

Correction method= # Reported T Limits: Tmin=0.573 Tmax=0.897
AbsCorr = ANALYTICAL

Data completeness= 0.993   Theta(max)= 76.686

R(reflections)= 0.0346(2294)   wR2(reflections)= 0.0953(2388)

S = 1.057   Npar= 155

The following ALERTS were generated. Each ALERT has the format
test-name_ALERT_alert-type_alert-level.
Click on the hyperlinks for more details of the test.

Alert level G

```
PLAT142_ALERT_4_G s.u. on b - Axis Small or Missing .............    0.00004 Ang.
PLAT143_ALERT_4_G s.u. on c - Axis Small or Missing .............    0.00015 Ang.
PLAT432_ALERT_2_G Short Inter X...Y Contact  C14      ..C14      .       3.16 Ang.
                                    1/2-x,3/2-y,3/2-z  =      7_566 Check
PLAT912_ALERT_4_G Missing # of FCF Reflections Above STh/L=  0.600         16 Note
PLAT953_ALERT_1_G Reported (CIF) and Actual (FCF) Hmax Differ by .          1 Units
PLAT969_ALERT_5_G The 'Henn et al.' R-Factor-gap value ..........       4.27 Note
            Predicted wR2: Based on SigI**2  2.23 or SHELX Weight  9.33
PLAT978_ALERT_2_G Number C-C Bonds with Positive Residual Density.          6 Info
```

0 **ALERT level A** = Most likely a serious problem - resolve or explain
0 **ALERT level B** = A potentially serious problem, consider carefully
0 **ALERT level C** = Check. Ensure it is not caused by an omission or oversight
7 **ALERT level G** = General information/check it is not something unexpected

1 ALERT type 1 CIF construction/syntax error, inconsistent or missing data
2 ALERT type 2 Indicator that the structure model may be wrong or deficient
0 ALERT type 3 Indicator that the structure quality may be low
3 ALERT type 4 Improvement, methodology, query or suggestion
1 ALERT type 5 Informative message, check

It is advisable to attempt to resolve as many as possible of the alerts in all categories. Often the minor alerts point to easily fixed oversights, errors and omissions in your CIF or refinement strategy, so attention to these fine details can be worthwhile. In order to resolve some of the more serious problems it may be necessary to carry out additional measurements or structure refinements. However, the purpose of your study may justify the reported deviations and the more serious of these should normally be commented upon in the discussion or experimental section of a paper or in the "special_details" fields of the CIF. checkCIF was carefully designed to identify outliers and unusual parameters, but every test has its limitations and alerts that are not important in a particular case may appear. Conversely, the absence of alerts does not guarantee there are no aspects of the results needing attention. It is up to the individual to critically assess their own results and, if necessary, seek expert advice.

Publication of your CIF in IUCr journals

A basic structural check has been run on your CIF. These basic checks will be run on all CIFs submitted for publication in IUCr journals (*Acta Crystallographica*, *Journal of Applied Crystallography*, *Journal of Synchrotron Radiation*); however, if you intend to submit to *Acta Crystallographica Section C* or *E* or *IUCrData*, you should make sure that full publication checks are run on the final version of your CIF prior to submission.

Publication of your CIF in other journals

Please refer to the *Notes for Authors* of the relevant journal for any special instructions relating to CIF submission.

PLATON version of 06/01/2024; check.def file version of 05/01/2024

Datablock iapp_utd1_final - ellipsoid plot

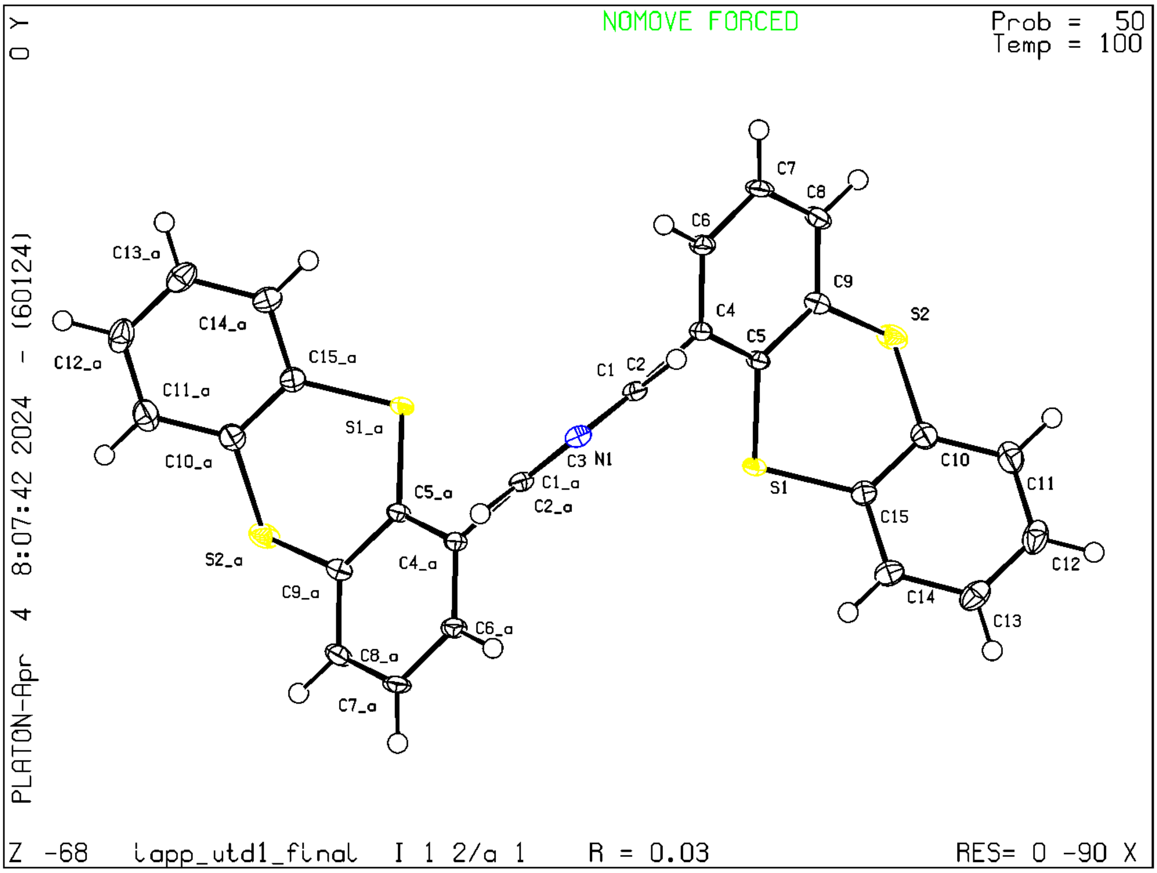

Supplement: Supplementary file 5 — Supplementary Data 2 [file 42004_2025_1620_MOESM5_ESM.pdf]
