# Supplementary material for: Systematic variation of the acceptor electrophilicity in donor-acceptor-donor emitters exhibiting efficient room temperature phosphorescence suited for digital luminescence
Source: Commun Chem. 2025 Sep 10;8:274. doi: 10.1038/s42004-025-01620-0 (PMC12423317; doi:10.1038/s42004-025-01620-0)

checkCIF/PLATON report

Structure factors have been supplied for datablock(s) iapp_utd3

THIS REPORT IS FOR GUIDANCE ONLY. IF USED AS PART OF A REVIEW PROCEDURE FOR PUBLICATION, IT SHOULD NOT REPLACE THE EXPERTISE OF AN EXPERIENCED CRYSTALLOGRAPHIC REFEREE.

No syntax errors found. CIF dictionary Interpreting this report

Datablock: iapp_utd3

Bond precision:	C-C = 0.0020 A		Wavelength=1.54184
Cell:	a=7.4913(2)	b=11.1972(3)	c=16.8013(5)
	alpha=106.090(2)	beta=93.322(2)	gamma=106.258(2)
Temperature:	100 K		

	Calculated	Reported
Volume	1285.85(7)	1285.85(6)
Space group	P -1	P -1
Hall group	-P 1	-P 1
Moiety formula	C28 H16 N2 S4, C H2 Cl2	C H2 Cl2, C28 H16 N2 S4
Sum formula	C29 H18 Cl2 N2 S4	C29 H18 Cl2 N2 S4
Mr	593.59	593.59
Dx,g cm-3	1.533	1.533
Z	2	2
Mu (mm-1)	5.494	5.494
F000	608.0	608.0
F000’	613.28	
h,k,lmax	9,14,21	9,14,20
Nref	5432	5349
Tmin,Tmax	0.446,0.610	0.298,0.605
Tmin’	0.269	

Correction method= # Reported T Limits: Tmin=0.298 Tmax=0.605
AbsCorr = ANALYTICAL

Data completeness= 0.985 Theta(max)= 76.807

R(reflections)= 0.0295(4975) wR2(reflections)= 0.0755(5349)

S = 1.045 Npar= 334

The following ALERTS were generated. Each ALERT has the format
test-name_ALERT_alert-type_alert-level.
Click on the hyperlinks for more details of the test.

Alert level C

```
PLAT042_ALERT_1_C Calc. and Reported MoietyFormula Strings  Differ     Please Check
              Calc: C28 H16 N2 S4, C H2 Cl2
              Rep.: C H2 Cl2, C28 H16 N2 S4
PLAT911_ALERT_3_C Missing FCF Refl Between Thmin & STh/L=    0.600           2 Report
                2-13  1,  -2 -1 17,
```

Alert level G

```
PLAT154_ALERT_1_G The s.u.'s on the Cell Angles are Equal ..(Note)      0.002 Degree
PLAT912_ALERT_4_G Missing # of FCF Reflections Above STh/L=  0.600         74 Note
PLAT941_ALERT_3_G Average HKL Measurement Multiplicity ..........        2.6 Low
PLAT969_ALERT_5_G The 'Henn et al.' R-Factor-gap value ..........       2.52 Note
              Predicted wR2: Based on SigI**2  3.00 or SHELX Weight  7.47
PLAT978_ALERT_2_G Number C-C Bonds with Positive Residual Density.          16 Info
```

0 **ALERT level A** = Most likely a serious problem - resolve or explain
0 **ALERT level B** = A potentially serious problem, consider carefully
2 **ALERT level C** = Check. Ensure it is not caused by an omission or oversight
5 **ALERT level G** = General information/check it is not something unexpected

2 ALERT type 1 CIF construction/syntax error, inconsistent or missing data
1 ALERT type 2 Indicator that the structure model may be wrong or deficient
2 ALERT type 3 Indicator that the structure quality may be low
1 ALERT type 4 Improvement, methodology, query or suggestion
1 ALERT type 5 Informative message, check

It is advisable to attempt to resolve as many as possible of the alerts in all categories. Often the minor alerts point to easily fixed oversights, errors and omissions in your CIF or refinement strategy, so attention to these fine details can be worthwhile. In order to resolve some of the more serious problems it may be necessary to carry out additional measurements or structure refinements. However, the purpose of your study may justify the reported deviations and the more serious of these should normally be commented upon in the discussion or experimental section of a paper or in the "special_details" fields of the CIF. checkCIF was carefully designed to identify outliers and unusual parameters, but every test has its limitations and alerts that are not important in a particular case may appear. Conversely, the absence of alerts does not guarantee there are no aspects of the results needing attention. It is up to the individual to critically assess their own results and, if necessary, seek expert advice.

Publication of your CIF in IUCr journals

A basic structural check has been run on your CIF. These basic checks will be run on all CIFs submitted for publication in IUCr journals (*Acta Crystallographica*, *Journal of Applied Crystallography*, *Journal of Synchrotron Radiation*); however, if you intend to submit to *Acta Crystallographica Section C* or *E* or *IUCrData*, you should make sure that full publication checks are run on the final version of your CIF prior to submission.

Publication of your CIF in other journals

Please refer to the *Notes for Authors* of the relevant journal for any special instructions relating to CIF submission.

PLATON version of 06/01/2024; check.def file version of 05/01/2024

Datablock iapp_utd3 - ellipsoid plot

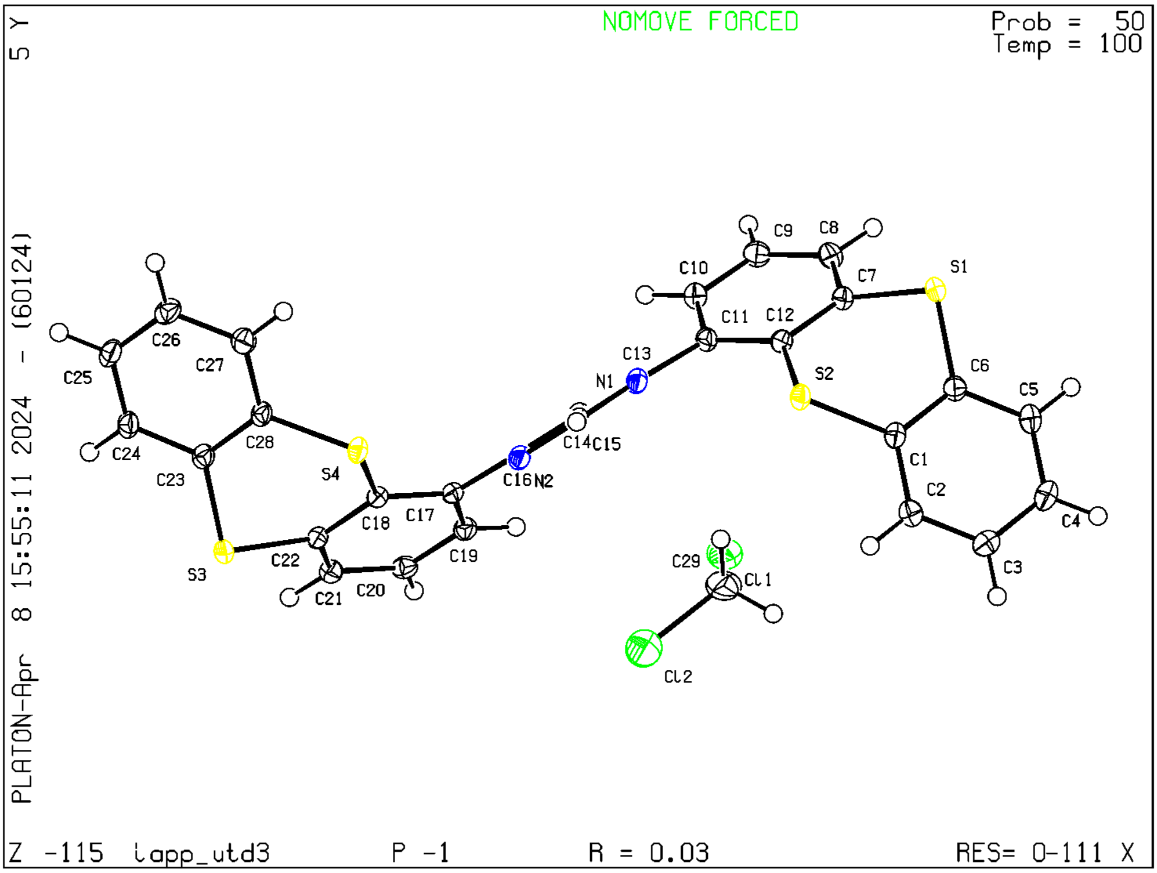

Supplement: Supplementary file 7 — Supplementary Data 4 [file 42004_2025_1620_MOESM7_ESM.pdf]
